# A systematic review of radiomics in giant cell tumor of bone (GCTB): the potential of analysis on individual radiomics feature for identifying genuine promising imaging biomarkers

**DOI:** 10.1186/s13018-023-03863-w

**Published:** 2023-06-07

**Authors:** Jingyu Zhong, Yue Xing, Guangcheng Zhang, Yangfan Hu, Defang Ding, Xiang Ge, Zhen Pan, Qian Yin, Huizhen Zhang, Qingcheng Yang, Huan Zhang, Weiwu Yao

**Affiliations:** 1 Department of Imaging, Tongren Hospital, Shanghai Jiao Tong University School of Medicine, Shanghai, 200336 China; 2 Department of Sports Medicine, Shanghai Sixth People’s Hospital, Shanghai Jiao Tong University School of Medicine, Shanghai, 200233 China; 3 Department of Orthopedics, Tongren Hospital, Shanghai Jiao Tong University School of Medicine, Shanghai, 200336 China; 4 Department of Pathology, Shanghai Sixth People’s Hospital, Shanghai Jiao Tong University School of Medicine, Shanghai, 200233 China; 5 Department of Orthopedics, Shanghai Sixth People’s Hospital, Shanghai Jiao Tong University School of Medicine, Shanghai, 200233 China; 6 Department of Radiology, Ruijin Hospital, Shanghai Jiao Tong University of Medicine, Shanghai, 200025 China

**Keywords:** Giant cell tumor of bone, Radiomics, Machine learning, Differential diagnosis, Quality improvement, Systematic review

## Abstract

**Purpose:**

To systematically assess the quality of radiomics research in giant cell tumor of bone (GCTB) and to test the feasibility of analysis at the level of radiomics feature.

**Methods:**

We searched PubMed, Embase, Web of Science, China National Knowledge Infrastructure, and Wanfang Data to identify articles of GCTB radiomics until 31 July 2022. The studies were assessed by radiomics quality score (RQS), transparent reporting of a multivariable prediction model for individual prognosis or diagnosis (TRIPOD) statement, checklist for artificial intelligence in medical imaging (CLAIM), and modified quality assessment of diagnostic accuracy studies (QUADAS-2) tool. The radiomic features selected for model development were documented.

**Results:**

Nine articles were included. The average of the ideal percentage of RQS, the TRIPOD adherence rate and the CLAIM adherence rate were 26%, 56%, and 57%, respectively. The risk of bias and applicability concerns were mainly related to the index test. The shortness in external validation and open science were repeatedly emphasized. In GCTB radiomics models, the gray level co-occurrence matrix features (40%), first order features (28%), and gray-level run-length matrix features (18%) were most selected features out of all reported features. However, none of the individual feature has appeared repeatably in multiple studies. It is not possible to meta-analyze radiomics features at present.

**Conclusion:**

The quality of GCTB radiomics studies is suboptimal. The reporting of individual radiomics feature data is encouraged. The analysis at the level of radiomics feature has potential to generate more practicable evidence for translating radiomics into clinical application.

**Supplementary Information:**

The online version contains supplementary material available at 10.1186/s13018-023-03863-w.

## Introduction

Giant cell tumor of bone (GCTB) is typically composed of neoplastic mononuclear stromal cells, macrophages and osteoclast-like giant cells [1], and marked by a mutation in the *H3F3A* gene [2]. GCTB has a potential of aggressive behavior with high local recurrence rate, and thus, needs personalized stratified management [3, 4]. Yet, GCTB rarely metastases to distinct site or shows malignant transformation [5]. Imaging is of importance throughout the clinical routine of GCTB management [5, 6], from differential diagnosis [7, 8], evaluation of response to denosumab [9], and prediction of local recurrence [10]. Radiomics, an emerging workflow that associates quantitative imaging biomarkers with significant clinical outcomes [11-15], has been employed in musculoskeletal oncology [16-19]. The radiomics models have also showed promising performance for diagnostic, predictive, and prognostic purpose in GCTB patients [20-28]. However, the quality of radiomics studies on GCTB has not been evaluated, and it is still unclear which radiomics features are genuinely of significance with biologic correlation.

As a subset of artificial intelligence, many recently developed tools have been recommended to assess the quality and reporting of radiomics research [18, 19, 29, 30], including radiomics quality score (RQS) [31], the transparent reporting of a multivariable prediction model for individual prognosis or diagnosis (TRIPOD) checklist [32], the checklist for artificial intelligence in medical imaging (CLAIM) [33], and the modified quality assessment of diagnostic accuracy studies (QUADAS-2) tool [34]. Although these tools are useful in identifying the reporting disadvantages, methodological shortness, and potential risk of bias in radiomics studies, their rating are all at the level of study. The impact factor of radiomics reproducibility has been measured at the level of radiomics features [35], while the approach of analysis at the level of radiomics feature has not been established so far, neither has the analysis on effect size of individual features been performed yet. Nevertheless, it is believed that genuinely promising biomarkers appear in multiple studies [36, 37], and the meta-analysis of these repeatably appearing features allows a signal of whether a predictor has genuine promise [38]. Therefore, we hypothesized that analysis at the level of radiomics features can provide additional information for radiomics studies.

The aim of the present study is to systematically assess the quality of radiomics research in GCTB and to test the feasibility of analysis at the level of radiomics feature.

## Materials and methods

### Protocol and workflow

Ethics committee approval is not required, because the nature of this study, which is a systematic review. This systematic review was conducted per Preferred Reporting Items for Systematic Reviews and Meta-Analyses (PRISMA) statement [39], and corresponding PRIMSA checklists are presented as Additional file 2. The review protocol has been been registered as CRD42022185399 via the International Prospective Register Of Systematic Reviews (PROSPERO; https://www.crd.york.ac.uk/prospero), and is present in Additional file 1: Note S1 and Additional file 3. The literature search, study selection, data extraction, quality assessment, and data analysis were duplicated by two independent reviewers each with 4 years’ experience in radiology and radiomics research. The disagreements were solved after consulting a third reviewer from our review group consisting of radiologists, orthopedists, and pathologists.

### Literature search and selection

We searched five peer-reviewed databases (PubMed, Embase, Web of Science, China National Knowledge Infrastructure, and Wanfang Data) until 31 July 2022 for primary research articles concerning on radiomics in GCTB for diagnostic, prognostic, or predictive purposes. We did not set publication period restrictions, while only articles in English, Japanese, Chinese, German or French were available. The titles and abstracts were screened after the removal of duplications. The full-texts and corresponding supplementary materials of these potential records were obtained to determine their eligibility. The reference lists of included articles were browsed by hand for additional potentially eligible articles. The search and selection strategy are shown in Additional file 1: Note S2.

### Data extraction and quality assessment

We used a data collection instrument to collect bibliographical information, study characteristics, radiomics considerations, and model metrics of included studies (Additional file 1: Table S1) [18, 19]. The included studies were comprehensively evaluated using RQS [31], TRIPOD [32], CLAIM [33], and QUADAS-2 tools [34] (Additional file 1: Tables S2 to S5). The RQS rating is a consensus list composed of six key domains with sixteen items emphasizing radiomics-specific issues, and is one of the most acceptable quality evaluation tools for radiomics researches [29, 30]. The TRIPOD statement provides a checklist consisting of twenty-two criteria with thirty-seven items, and is recommended for distinguishing shortness of model reporting of radiomics models [29, 30]. The CLAIM tool includes seven topics with forty-two items, and is considered as a better tool to identify technical disadvantages in radiomics studies [18]. The QUADAS-2 tool was tailored to our review by modifying the signaling questions [18, 19]. The consensus reached during data extraction and quality assessment are shown in Additional file 1: Note S3.

### Data synthesis and analysis

The statistical analysis was performed with R language version 4.1.3 (https://www.r-project.org/) within RStudio version 1.4.1106 (https://www.rstudio.com/) [40]. The RQS rating, the ideal percentage of RQS, and adherence rates of RQS, TRIPOD and CLIAM were calculated. In case a score of at least one point for each item was obtained without minus points, it was considered to have basic adherence, as those have been reported [18, 19, 29, 30]. The QUADAS-2 assessment result was summarized. A two-tailed *P* value < 0.05 indicated statistical significance, unless otherwise specified. In current review, we performed an analysis at the level of radiomics feature. We determined the group of radiomics features in GCTB models, and find out whether they appeared in multiple studies [36-38]. The meta-analysis was not conducted due to the high heterogeneity and insufficient reporting [41]. We further determined the model type [32] and study phase [42] to show the gap between current studies and clinical application (Additional file 1: Tables S6 and S7). The detailed data analysis method is described in Additional file 1: Note S4.

## Results

### Literature search

**Fig. 1:**
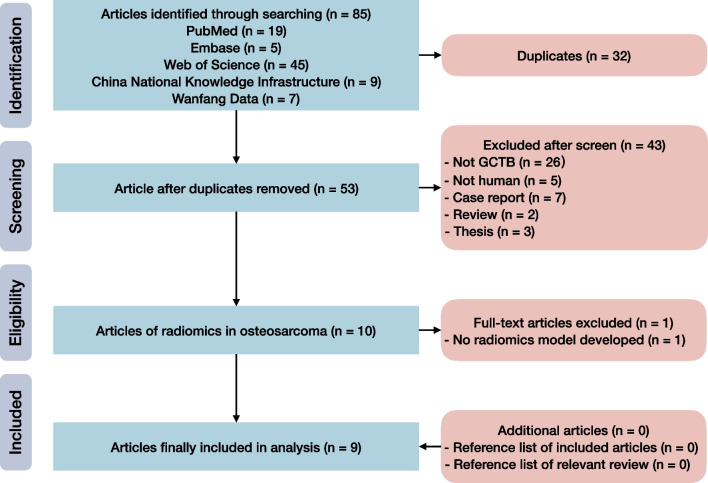
Flow diagram of study inclusion

Our systematic review identified 53 unique records after removal of 32 duplicates (Fig. 1). We screened their titles and abstracts, and obtained the full-texts and Additional file 1 of ten potentially available articles for eligibility assessment. Finally, nine articles were included [20-28]. There were no additional eligible articles detected by browsing reference lists of included articles and relevant reviews.

### Study characteristics

**Fig. 2:**
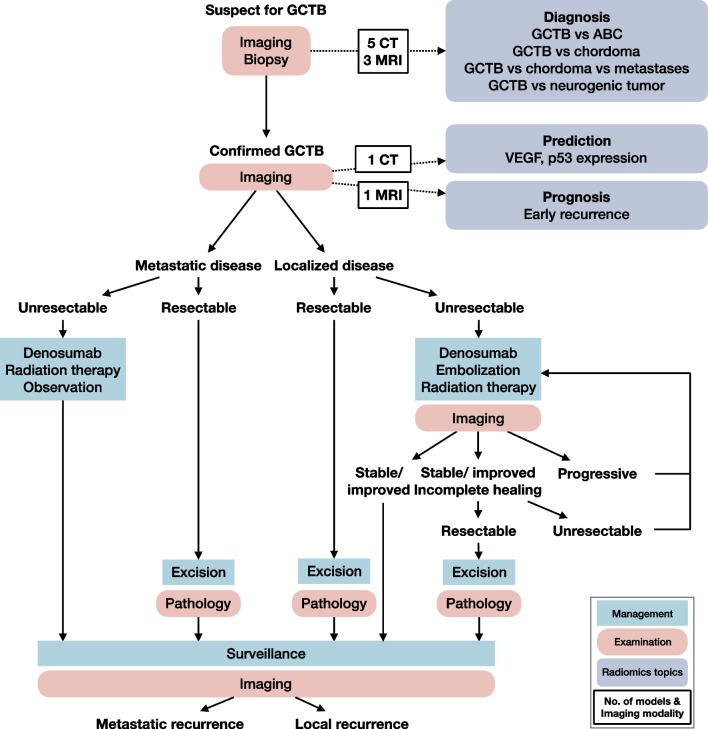
Imaging and radiomics in GCTB management

The characteristics of included studies was summarized (Table 1). The average ± standard deviation (median, range) sample size of the included studies was of 97 ± 56 (92, 29–215). Five studies were based on CT [20-22, 25, 28], three were conducted with MRI [23, 24, 26], respectively, and the left one study used both CT and MRI [27]. The included nine articles covered a vast range of clinical questions of GCTB (Fig. 2). Seven models attempted to differentiate GCTB from other types of tumors, including aneurysmal bone cyst [21, 24], chordoma [25-27], neurogenic tumor [28], or metastatic tumor [26], but only one model compared the performance of radiomics with radiologists’ assessment and showed significant improvement [24]. One model was developed for expression of *p53* and *VEGF* in GCTB, and provided better performance than clinical scoring or staging system [23]. One model was built for prognostic purpose for early recurrence of spinal GCTB [22].

**Table 1:** Characteristics of included studies

Study	Sample size	Imaging modality	Comparing test	Reference standard	Biomarker	Outcome	RQS (%)	TRIPOD (%)	CLAIM (%)
Nie [20]	92 (33 GCTB + 59 chordoma)	CT	None	Histology	Diagnosis	GCTB vs. chordoma	44	73	65
Shi [21]	43 (34 GCTB + 9 ABC)	CT	None	Histology	Diagnosis	GCTB vs. ABC	0	38	35
Wang [22]	62 GCTB	CT	None	Follow up	Prognosis	Early recurrence in spinal GCTB	17	65	59
Wang [23]	80 GCTB	MRI	SINS score, Enneking stage	Immunohistochemical staining	Prediction	Expression of *p53* and *VEGF* in GCTB	25	65	61
Wu [24]	29 (16 GCTB + 13 ABC)	MRI	Radiologists’ assessment	Histology	Diagnosis	GCTB vs. ABC	25	62	51
Yin [25]	95 (42 GCTB + 53 chordoma)	CT	None	Histology	Diagnosis	GCTB vs. chordoma in sacrum	31	58	67
Yin [26]	120 (30 GCTB + 54 + chordoma + 36 metastatic tumors)	MRI	None	Histology	Diagnosis	GCTB vs. chordoma vs. metastatic tumor in sacrum	31	58	57
Yin [27]	137 (54 GCTB + 83 chordoma)	CT + MRI	None	Histology	Diagnosis	GCTB vs. chordoma in sacrum	36	62	61
Yin [28]	215 (120 GCTB + 95 neurogenic tumors)	CT	None	Histology	Diagnosis	GCTB vs. neurogenic tumor in pelvic and sacral tumor	0	65	59

*ABC* aneurysmal bone cyst, *CLAIM* checklist for artificial intelligence in medical imaging, *GCTB* giant cell tumor of bone, *RQS* radiomics quality score, *SINS* spinal instability neoplastic score, *TRIPOD* transparent reporting of a multivariable prediction model for individual prognosis or diagnosis

The radiomics models was established with various methodologic settings (Table 2). Most of the models manually defined the region of interest (89%), by radiologists with relevant subspecialist expertise (44%) or unspecified expertise (44%). Seven models used intraclass coefficient to measure the reproducibility of radiomics features extracted from two segmentations, and selected the reproducible ones. Artificial Intelligence Kit were employed in more than a half of the models for feature extraction (55%), while less than a half of the models include non-radiomics feature into the model (44%). According to the sample size and the validation datasets, one model was defined as TRIPOD type 3 model, and four models were classified as phase II for image mining. The details of studies and models are present in Additional file 1: Table S8 to S11.

**Table 2:** Radiomics analysis details of included studies

Study	Segmentation and software	Observers and agreement	Feature extraction software	Non-radiomics features	Validation dataset	Model type	Phase classification
Nie [20]	Manual; ITK-SNAP	2 URs; ICC	Radiomics cloud platform	Clinical parameters	Separate data from other institution	3	1
Shi [21]	Manual; Image J	Not documented	Image J	None	Exactly the same data	1a	0
Wang [22]	Manual; not documented	2 SRs; none	Pyradiomics	None	Tenfold cross-validation	1b	0
Wang [23]	Manual; Image J	2 SRs; none	Pyradiomics	Clinical parameters	Tenfold cross-validation	1b	0
Wu [24]	Manual; ITK-SNAP	2 URs; ICC	Artificial intelligence kit	Radiologists’ assessment	Exactly the same data	1a	0
Yin [25]	Manual; ITK-SNAP	2 SRs; ICC	Artificial intelligence kit	None	Randomly splitting data	2a	0
Yin [26]	Manual; ITK-SNAP	2 URs; ICC	Artificial intelligence kit	None	Randomly splitting data	2a	1
Yin [27]	Manual; ITK-SNAP	2 SRs; ICC	Artificial intelligence kit	Clinical parameters	Randomly splitting data	2a	1
Yin [28]	Semi-automatic; MITK	2 SRs; ICC	Artificial intelligence kit	Clinical parameters	Randomly splitting data	2a	1

*ICC* intraclass coefficient, *SR* radiologist with relevant subspecialist expertise, *UR* radiologist with unspecified expertise

### Study quality

**Fig. 3:**
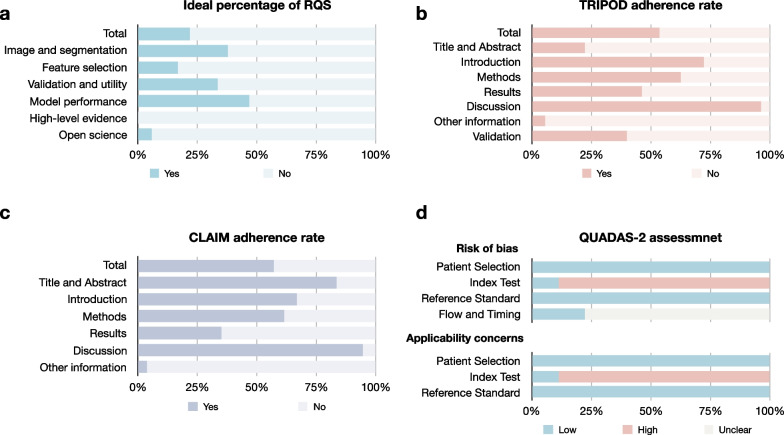
Quality assessment of included studies. **a** ideal percentage of RQS, **b** TRIPOD adherence rate, **c** CLAIM adherence rate **d** QUADAS-2 assessment result

The overall quality of GCTB radiomics studies was suboptimal (Fig. 3). The average ± standard deviation (median, range) of the total RQS rating was 9.3 ± 5.1 (11, − 2 to 16) and a percentage of ideal score of 26% (9.3/36) (Table 3). The overall adherence rate of RQS, TRIPOD and CLAIM were 45% (65/144), 56% (142/252), and 57% (262/459), respectively (Tables 3, 4 and 5). The risk of bias and applicability concerns were mainly related to the index test, because the models were not validated using independent external datasets. The quality ratings per study are present in Additional file 1: Table S12 to S15.

**Table 3:** RQS rating of included studies

16 Items according to 6 key domains	Range	Median (range)	Percentage of ideal score, n (%)	Adherence rate, n (%)
Total 16 items	**− 8 to 36**	**11 (− 2 to 16)**	**9.3 (26)**	**65/144 (45)**
Domain 1: protocol quality and stability in image and segmentation	**0 to 5**	**2 (1 to 2)**	**1.9 (38)**	**17/36 (47)**
Protocol quality	0 to 2	1 (1 to 1)	1.0 (50)	9/9 (100)
Multiple segmentations	0 to 1	1 (0 to 1)	0.9 (89)	8/9 (89)
Test–retest	0 to 1	0 (0 to 0)	0.0 (0)	0/9 (9)
Phantom study	0 to 1	0 (0 to 0)	0.0 (0)	0/9 (0)
Domain 2: feature selection and validation	**− 8 to 8**	**5 (− 8 to 6)**	**1.3 (17)**	**13/18 (72)**
Feature reduction or adjustment of multiple testing	− 3 to 3	3 (− 3 to 3)	2.3 (78)	8/9 (89)
Validation	− 5 to 5	2 (− 5 to 3)	− 1.0 (0)	5/9 (56)
Domain 3: biologic/clinical validation and utility	**0 to 6**	**3 (2 to 6)**	**3.6 (59)**	**21/36 (58)**
Non-radiomics features	0 to 1	1 (0 to 1)	0.6 (56)	5/9 (56)
Biologic correlations	0 to 1	1 (0 to 1)	0.6 (56)	5/9 (56)
Comparison to “gold standard”	0 to 2	0 (0 to 2)	0.4 (22)	2/9 (22)
Potential clinical utility	0 to 2	2 (2 to 2)	2.0 (100)	9/9 (100)
Domain 4: model performance index	**0 to 5**	**2 (1 to 4)**	**2.3 (47)**	**12/27 (44)**
Cut-off analysis	0 to 1	0 (0 to 0)	0.0 (0)	0/9 (0)
Discrimination statistics	0 to 2	2 (1 to 2)	1.7 (83)	9/9 (100)
Calibration statistics	0 to 2	0 (0 to 2)	0.7 (33)	3/9 (33)
Domain 5: high level of evidence	**0 to 8**	**0 (0 to 0)**	**0.0 (0)**	**0/18 (0)**
Prospective study	0 to 7	0 (0 to 0)	0.0 (0)	0/9 (0)
Cost-effectiveness analysis	0 to 1	0 (0 to 0)	0.0 (0)	0/9 (0)
Domain 6: open science and data	**0 to 4**	**0 (0 to 1)**	**0.2 (6)**	**2/9 (22)**

The ideal score was described as score and percentage of score to ideal score for each item. In the cases where a score of one point per item was obtained, the study was considered to have basic adherence to each item. The adherence rate was calculated as proportion of the number of articles with basic adherence to number of total articles. The bolded numbers indicated the sum of domains or RQS

*RQS* radiomics quality score

**Table 4:** TRIPOD adherence of included studies

37 Selected items in 22 criteria according to 7 sections (N = 9)	Study, n (%)
Overall (excluding items 5c, 11, 14b, 10c, 10e, 12, 13, 17, and 19a)	**142/252 (56)**
Section 1: Title and abstract	**4/18 (22)**
1. Title—identify developing/validating a model, target population, and the outcome	0/9 (0)
2. Abstract—provide a summary of objectives, study design, setting, participants, sample size, predictors, outcome, statistical analysis, results, and conclusions	4/9 (44)
Section 2: Introduction	**13/18 (72)**
3a. Background—explain the medical context and rationale for developing/validating the model	9/9 (100)
3b. Objective—specify the objectives, including whether the study describes the development/validation of the model or both	4/9 (44)
Section 3: Methods	**73/117 (62)**
4a. Source of data—describe the study design or source of data (randomized trial, cohort, or registry data)	9/9 (100)
4b. Source of data—specify the key dates	9/9 (100)
5a. Participants—specify key elements of the study setting including number and location of centers	9/9 (100)
5b. Participants—describe eligibility criteria for participants (inclusion and exclusion criteria)	8/9 (89)
5c. Participants—give details of treatment received, if relevant (N = 1)	1/1 (100)
6a. Outcome—clearly define the outcome, including how and when assessed	9/9 (100)
6b. Outcome—report any actions to blind assessment of the outcome	0/9 (0)
7a. Predictors—clearly define all predictors, including how and when assessed	8/9 (89)
7b. Predictors—report any actions to blind assessment of predictors for the outcome and other predictors	2/9 (22)
8. Sample size—explain how the study size was arrived at	0/9 (0)
9. Missing data—describe how missing data were handled with details of any imputation method	0/9 (0)
10a. Statistical analysis methods—describe how predictors were handled	9/9 (100)
10b. Statistical analysis methods—specify type of model, all model-building procedures (any predictor selection), and method for internal validation	8/9 (89)
10d. Statistical analysis methods—specify all measures used to assess model performance and if relevant, to compare multiple models (discrimination and calibration)	2/9 (22)
11. Risk groups—provide details on how risk groups were created, if done (N = 1)	1/1 (100)
Section 4: Results	**25/54 (46)**
13a. Participants—describe the flow of participants, including the number of participants with and without the outcome. A diagram may be helpful	3/9 (33)
13b. Participants—describe the characteristics of the participants, including the number of participants with missing data for predictors and outcome	3/9 (33)
14a. Model development—specify the number of participants and outcome events in each analysis	8/9 (89)
14b. Model development—report the unadjusted association between each candidate predictor and outcome, if done (N = 5)	1/1 (100)
15a. Model specification—present the full prediction model to allow predictions for individuals (regression coefficients, intercept)	3/9 (33)
15b. Model specification—explain how to the use the prediction model (nomogram, calculator, etc.)	2/9 (22)
16. Model performance—report performance measures (with confidence intervals) for the prediction model	6/9 (67)
Section 5: Discussion	**276/27 (96)**
18. Limitations—discuss any limitations of the study	8/9 (89)
19b. Interpretation—give an overall interpretation of the results	9/9 (100)
20. Implications—discuss the potential clinical use of the model and implications for future research	9/9 (100)
Section 6: Other information	**1/18 (6)**
21. Supplementary material—provide information about the availability of supplementary resources, such as study	0/9 (0)
22. Funding—give the source of funding and the role of the funders for the present study	1/9 (11)
Section 7: Validation for model type 2a, 2b, 3, and 4 (N = 5)	**8/20 (40)**
10c. Statistical analysis methods—describe how the predictions were calculated	2/5 (40)
10e. Statistical analysis methods—describe any model updating (recalibration), if done (N = 0)	n/a
12. Development versus validation—identify any differences from the development data in setting, eligibility criteria, outcome, and predictors	3/5 (60)
13c. Participants (for validation)—show a comparison with the development data of the distribution of important variables	3/5 (60)
17. Model updating—report the results from any model updating, if done (N = 0)	n/a
19a. Interpretation (for validation)—discuss the results with reference to performance in the development data and any other validation data	0/5 (0)

In the cases where a score of one point per item was obtained, the study was considered to have basic adherence to each item. The adherence rate was calculated as proportion of the number of articles with basic adherence to number of total articles. During the calculation, the “if done” or “if relevant” items (5c, 11, and 14b) and validation items (10c, 10e, 12, 13, 17, and 19a) were excluded from both the denominator and numerator. The bolded numbers indicated the sum of sections or TRIPOD

*TRIPOD* transparent reporting of a multivariable prediction model for individual prognosis or diagnosis, *n/a* not applicable

**Table 5:** CLAIM adherence of included studies

CLAIM items (N = 9)	Study, n (%)
Overall (excluding item 15a and 27)	**262/459 (57)**
Section 1: Title and abstract	**15/18 (83)**
1. Title or abstract—identification as a study of AI methodology	9/9 (100)
2. Abstract—structured summary of study design, methods, results, and conclusions	6/9 (67)
Section 2: Introduction	**18/27 (67)**
3. Background—scientific and clinical background, including the intended use and clinical role of the AI approach	9/9 (100)
4a. Study objective	9/9 (100)
4b. Study hypothesis	0/9 (0)
Section 3: Methods	**193/315 (61)**
5. Study design—prospective or retrospective study	9/9 (100)
6. Study design—study goal, such as model creation, exploratory study, feasibility study, non-inferiority trial	9/9 (100)
7a. Data—data source	9/9 (100)
7b. Data—data collection institutions	9/9 (100)
7c. Data—imaging equipment vendors	9/9 (100)
7d. Data—image acquisition parameters	9/9 (100)
7e. Data—institutional review board approval	7/9 (78)
7f. Data—participant consent	5/9 (56)
8. Data—eligibility criteria	8/9 (89)
9. Data—data pre-processing steps	1/9 (11)
10. Data—selection of data subsets (segmentation of ROI in radiomics studies)	8/9 (89)
11. Data—definitions of data elements, with references to Common Data Elements	9/9 (100)
12. Data—de-identification methods	0/9 (0)
13. Data—how missing data were handled	0/9 (0)
14. Ground truth—definition of ground truth reference standard, in sufficient detail to allow replication	9/9 (100)
15a. Ground truth—rationale for choosing the reference standard, if alternatives exist (N = 0)	n/a
15b. Ground truth—definitive ground truth	9/9 (100)
16. Ground truth—manual image annotation	5/9 (56)
17. Ground truth—image annotation tools and software	1/9 (11)
18. Ground truth—measurement of inter- and intra-rater variability; methods to mitigate variability and/or resolve discrepancies	6/9 (67)
19a. Data partitions—intended sample size and how it was determined	9/9 (100)
19b. Data partitions—provided power calculation	0/9 (0)
19c. Data partitions—distinct study participants	3/9 (33)
20. Data partitions—how data were assigned to partitions; specify proportions	3/9 (33)
21. Data partitions—level at which partitions are disjoint (e.g., image, study, patient, institution)	9/9 (100)
22a. Model—provided reproducible model description	8/9 (89)
22b. Model—provided source code	0/9 (0)
23. Model—software libraries, frameworks, and packages	5/9 (56)
24. Model—initialization of model parameters (e.g., randomization, transfer learning)	0/9 (0)
25. Training—details of training approach, including data augmentation, hyperparameters, number of models trained	8/9 (89)
26. Training—method of selecting the final model	7/9 (78)
27. Training—ensembling techniques, if applicable (N = 5)	5/5 (100)
28. Evaluation—metrics of model performance	9/9 (100)
29. Evaluation—statistical measures of significance and uncertainty (e.g., confidence intervals)	6/9 (67)
30. Evaluation—robustness or sensitivity analysis	1/9 (11)
31. Evaluation—Methods for explainability or interpretability (e.g., saliency maps), and HOW they were validated	2/9 (22)
32. Evaluation—validation or testing on external data	1/9 (11)
Section 4: Results	**19/54 (35)**
33. Data—flow of participants or cases, using a diagram to indicate inclusion and exclusion	3/9 (33)
34. Data—demographic and clinical characteristics of cases in each partition	3/9 (33)
35a. Model performance—test performance	5/9 (56)
35b. Model performance—benchmark of performance	2/9 (22)
36. Model performance—estimates of diagnostic accuracy and their precision (such as 95% confidence intervals)	6/9 (67)
37. Model performance—failure analysis of incorrectly classified cases	0/9 (0)
Section 5: Discussion	**17/18 (94)**
38. Study limitations, including potential bias, statistical uncertainty, and generalizability	8/9 (89)
39. Implications for practice, including the intended use and/or clinical role	9/9 (100)
Section 6: Other information	**1/27 (4)**
40. Registration number and name of registry	0/9 (0)
41. Where the full study protocol can be accessed	0/9 (0)
42. Sources of funding and other support; role of funders	1/9 (11)

In the cases where a score of one point per item was obtained, the study was considered to have basic adherence to each item. The adherence rate was calculated as proportion of the number of articles with basic adherence to number of total articles. During the calculation, the “if alternatives exist” item (15a) and “if applicable” item (27) were excluded from both the denominator and numerator. The bolded numbers indicated the sum of sections or CLAIM

*CLAIM* checklist for artificial intelligence in medical imaging

The RQS rating assessed the studies from a radiomics-specific view, pointing out the deficiency in test–retest (0%), phantom study (0%), cut-off analysis (0%), and cost-effective analysis (0%). The TRIPOD checklist showed room for improvement in reporting of title (0%), and blindness of outcome and predictor assessment (0% and 0%). The CLAIM tool identified shortness in technical aspects including study hypothesis statement (0%), data de-identification method (0%), and failure analysis (0%). The disadvantage of comparing test (22% and 22%) drew attention of the RQS rating and the CLAIM tool, while the lacking of sample size determination with power calculation (0% and 0%) and missing data handling (0% and 0%) were both addressed by the TRIPOD checklist and the CLAIM tool. The validation (0%, 40% and 11%) and open science (6%, 6%, and 4%) were emphasized by all three tools.

### Analysis at the level of radiomic feature

**Fig. 4:**
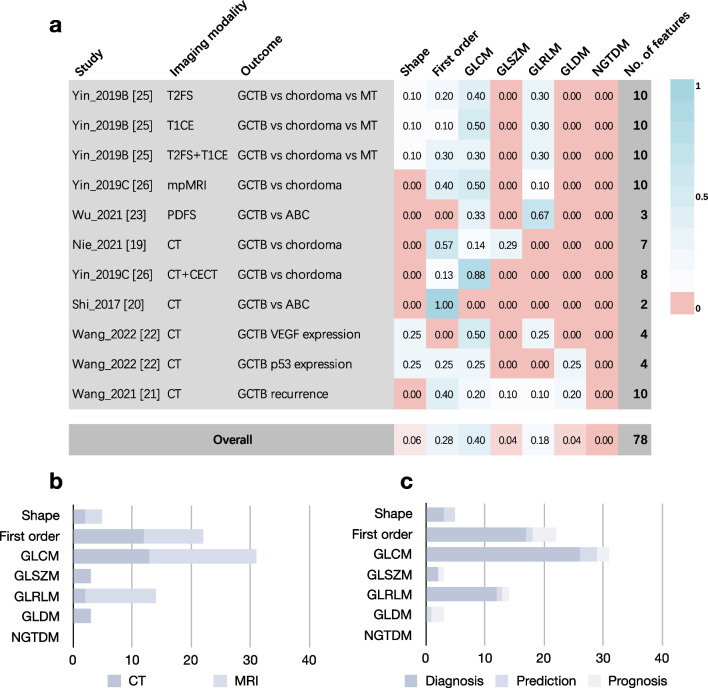
The selected radiomic features in models. *T2FS* T2-weighted imaging with fat saturation, *T1CE* T1-weighted imaging with contrast-enhancement, *mpMRI* multiparametric MRI (T1WI, T2WI, DWI, and T1CE), *PDFS* proton-density-weighted imaging with fat saturation, *CECT* contrast-enhanced CT, *GLCM* gray level concurrence matrix, *GLSZM* gray level size zone matrix, *GLRLM* gray level run length matrix, *GLDM* gray level dependence matrix, *NGTDM* neighbourhood gray tone difference matrix

The radiomics features selected for model building were summarized (Fig. 4). The multiple models developed in the same study were counted as different models [23, 26, 27] and one study did not document the selected features were excluded [25]. The gray level co-occurrence matrix features (40%), first order features (28%), and gray-level run-length matrix features (18%) were most selected features out of all reported features in GCTB radiomics. The gray level co-occurrence matrix features and first order features were usually selected in both CT-based (34% and 37%) and MRI-based (23% and 42%) models, but only gray-level run-length matrix features remained a percentage of 28% in MRI-based models. These three feature families also showed high percentages of included features in diagnostic models (28%, 43%, and 20%). In contrast, none of the neighbourhood gray-tone difference matrix features was considered of significance for radiomics model. Notably, none of the reported individual feature has appeared repeatably in multiple studies, although some of them attempted to answer the same clinical question in GCTB.

## Discussion

This review found that most of the current GCTB radiomics researches developed diagnostic models. Their methodological and reporting quality was suboptimal according to the RQS rating, the TRIPOD checklist, and the CLAIM tool. The risk of bias related to index testing has been identified by the QUADAS-2 tool. The most three significant feature families in GCTB radiomics models were gray level co-occurrence matrix (GLCM) features, first order features, and gray-level run-length matrix (GLRLM) features.

Our review identified seven out of nine studies that aimed to distinguish GCTB from other tumors. The differentiation between GCTB and aneurysmal bone cyst may be difficult, when the GCTB contains obvious cystic component or formats secondary aneurysmal bone cysts [43]. Most of GCTB develop in long bones, while it may mimic chordoma when it occurs in sacrum [44]. The studies claimed that radiomics models could offer a valuable contribution to the differential diagnosis [21, 24-28], while it is still unclear whether the radionics could provide better performance comparing to the radiologists [24]. Further, the definitive diagnosis is required for the malignant GCTB cannot be differentiated radiologically and histopathology [45]. Pitiably, none of the GCTB radiomics research investigated the vital differential diagnosis between the malignant GCTB and the conventional GCTB. One radiomics model pre-operatively predicted the expression of *p53* and *VEGF* in GCTB, and showed better performance than current methods [23]. Since the mutant of *p53* and high expression of *VEGF* have been considered as risk factors for local recurrence and malignant transformation in GCTB [46-48], the prediction has potential in choosing optimal treatment selections and surveillance protocols [23]. However, as an established targeted therapy for GCTB [10], the predictive model for GCTB response to denosumab has not been built yet, only a radiomics analysis on radiography showed changes of feature readouts during treatment [49]. There was only one prognostic radiomics model developed for early recurrence of the spinal GCTB [22]. Considering the complex treatment procedure of GCTB [3, 4], the prognostic models are of urgent to improve management strategies.

The insufficient study quality of radiomics studies has been repeatedly addressed [16-19, 29, 30, 50]. The ideal percentage of the RQS rating of GCTB radiomics researches was comparable to other musculoskeletal sarcomas [16-19]. The adherence rate of the TRIPOD checklist and the CLAIM tool were also similar to previous reviews [18, 19, 29, 30, 50]. The prospective study design, phantom study, test–retest analysis, validation, analysis of cut-offs, cist-effectiveness and clinical utility, as well as open science items have been suggested as common issues across radiomics research. However, the RQS includes five steps in the radiomics workflow: data selection, medical imaging, feature extraction, exploratory analysis, and modelling [13, 31]. We supposed that some of the issue may not be possible in one single article that aimed to develop and validate a model, but can be accomplished in a series of articles that aimed to identify the robust radiomics features, to tell whether the model is possible, and to test the model in the real-world, respectively. In spite of the suboptimal methodological quality itself, it could be another reasonable cause for low RQS rating of current modeling articles. Actually, a checklist specialized for radiomics robustness researches has been already developed [51], and there are other guidelines could be employed for radiomics investigations in clinical settings [52-55]. In contrast, the TRIPOD checklist and the CLAIM tool might be more suitable for current modeling radiomics researches, because they were designed for quality evaluation at the level of model. The TRIPOD checklist and the CLAIM tool can both identify disadvantages in missing data handling and sample size or power calculation, while the CLAIM can better capture unique shortness in radiomics researches, such as data de-identification and failure analysis [17]. The benefit of CLAIM has been also confirmed in our review that it could provide more technical insights for study design and reporting. The Image Biomarkers Standardization Initiative (IBSI) checklist is another potentially available tool for radiomics research [56]. We did not apply the IBSI checklist since it is largely overlapping with the RQS, the TRIPOD, and the CLAIM. The TRIPOD checklist and the QUADAS tool with artificial intelligence extensions is now under development, it would be interesting to test their feasibility in radiomics modeling researches [57, 58].

The meta-analysis was not possible neither at the level of study nor at the level of radiomics feature. Nevertheless, we summarized the feature family of the selected features, and identified three most important families. The radiomics researches are commonly haphazard, inconsistent, and underpowered, with most appearing promising due to methodological error rather than intrinsic ability [36, 37], For avoiding biases and pitfalls introduced during the design, analysis, or reporting, there were approaches described at the level of study [59]. Although Kothari et al. have tried to summarized the repeatedly appearing features in prognostic models of non-small cell lung cancer [60], this is the first attempt for meta-analyzing the repeatably appearing features so far. We believe this approach could allow us to tell whether an imaging biomarker has genuine promise [36-38]. Unfortunately, this approach is currently hindered by insufficient reporting of effect size of individual radiomics features, and the limited number of studies. Although the association between each candidate predictor and outcome (item 14b) has been addressed as an “if done” item in the TRIPOD checklist, it is seldomly done in radiomics researches. It is not reasonable to report the effect size of all tested radiomics features, but at least the reporting of the effect size of the selected radiomics features is encouraged in the future. Except for identifying meaningful features, this approach can guide future investigation in radiomics robustness and biological correlation. The current radiomics robustness analysis weighs each radiomics feature equal since they all potentially correlate with clinical outcomes. Instead of testing a huge amount of radiomics features, the number of features that needed to be test could be lessen to those with clinically significance [51, 61]. The radiomics workflow for specific clinical purpose could be simplified, because only a limited number of features needed to be robust. The data-driven radiomics processes extract features with no a priori assumptions on their correlation with biological processes, but the biological links could be explored a posteriori [61, 62]. Comparing to the features without clinical meaning, those associated with subsequent outcomes have a higher possibility to correlate with specific biological processes and pathways.

Our review has several limitations that should be acknowledged. Firstly, there were only a limited number of articles included in our review, but our review focused on the GCTB to provide insights for this field. There were some studies from the same institutions [22, 23, 25-27], which potentially influenced on the results of the current systematic review and introduced bias. GCTB occurs most frequently in the long bones of the extremities, but it is notable that six out of nine included studies focused on tumors of axial bones [20, 22, 23, 25-27].We did not include the GCTB researches using deep learning methodology, because one of our study aims was to test the feasibility of analysis at the level of radiomics feature. Secondly, the study quality was assessed by multiple tools, including the RQS, the TRIPOD, and the CLAIM, as these three tools have been confirmed to be suitable for radiomics reviews [18, 19, 29, 30, 50]. However, some items and their weight in the evaluation still needs clarification [18, 63]. The CheckList for EvaluAtion of Radiomics research (CLEAR) has been developed to improve the quality and reliability and, in turn, the reproducibility of radiomics research [64]. This tool may serve well as a single and complete scientific documentation tool for authors and reviewers to improve the radiomics literature.However, we did not utilize it, since this checklist has not been introduced to the radiomics community when the current systematic review was undergoing. We are going to use this tool in future researches and reviews. Thirdly, the meta-analysis at the level of radiomics features was not performed due to the limited number of studies and suboptimal reporting of effect size of individual radiomics features. Our group introduced this approach here, and plan to test its feasibility in other diseases which have been more widely investigated. The selection of radiomics features strongly depends on the model used [65]. Since statistically similar models may generally identify different features as relevant, the selection of radiomics features by a single model is misleading. Hence, there is a need for determining whether features are biologically relevant imaging biomarkers. The meta-analysis on repeatedly appearing features in multiple models might be possible, when a sufficient number of models have been established with complete reporting for a similar clinical question [15, 66]. Lastly, the meta-analysis at the level of radiomics models has not been conducted because of the high heterogeneity of included studies. The meta-analysis could be done with evidence rating in an updated review, when there are reasonable number of models developed with homogeneity.

In conclusion, the methodological and reporting quality of GCTB radiomics studies is insufficient. More research for predictive and prognostic purpose are encouraged, and the quality of radiomics models distinguishing GCTB from other tumors needs improvement. The room for methodological improvement includes external validation, association with biological, analysis of clinical utility, and open science. The reporting of effect size of individual radiomics feature is necessary for identifying genuine promising imaging biomarkers.

## Supplementary Information

**Additional file 1.** Supplementary Methods and Results.

**Additional file 2.** PRISMA Checklists.

**Additional file 3.** PROSPERO Review Protocol.

## Data Availability

The datasets used and/or analyzed during the current study are available from the corresponding author on reasonable request.

## Abbreviations

CLAIM: CheckList for artificial intelligence in medical imaging
GCTB: Giant cell tumor of bone
RQS: Radiomics quality score
TRIPOD: Transparent reporting of a multivariable prediction model for individual prognosis or diagnosis
QUADAS-2: Modified quality assessment of diagnostic accuracy studies
