# Supplementary material for: A systematic review of radiomics in giant cell tumor of bone (GCTB): the potential of analysis on individual radiomics feature for identifying genuine promising imaging biomarkers
Source: J Orthop Surg Res. 2023 Jun 7;18:414. doi: 10.1186/s13018-023-03863-w (PMC10249293; doi:10.1186/s13018-023-03863-w)
Supplement: Supplementary file 1 — Additional file 1. Supplementary Methods and Results. [file 13018_2023_3863_MOESM1_ESM.docx]

**Supplementary Material**

**Title:** A systematic review of radiomics in giant cell tumor of bone (GCTB): the potential of analysis on individual radiomics feature for identifying genuine promising imaging biomarkers

**List of Supplementary Material**

Supplementary Note S1 Review protocol

Supplementary Note S2 Search strategy and study selection

Supplementary Note S3 Consensus reached during data extraction and quality assessment

Supplementary Note S4 Data synthesis and analysis methods

Supplementary Table S1 Data extraction instrument

Supplementary Table S2 RQS elements according to six key domains

Supplementary Table S3 TRIPOD reporting completeness checklist

Supplementary Table S4 CLAIM for authors and reviewers

Supplementary Table S5 QUADAS-2 tool for risk of bias and concern on application

Supplementary Table S6 Types of prediction model studies covered by the TRIPOD statement

Supplementary Table S7 Trials classifications for image mining tools development process

Supplementary Table S8 Study characteristics of included studies

Supplementary Table S9 PICOT of included studies

Supplementary Table S10 Radiomics methodological issue of included studies

Supplementary Table S11 Model presentation and performance metrics of included studies

Supplementary Table S12 RQS rating per study

Supplementary Table S13 TRIPOD adherence per study

Supplementary Table S14 CLAIM adherence per study

Supplementary Table S15 QUADAS-2 assessment per study

**Supplementary Note S1 Review protocol**

**Registration information**

First drafted date: 01/06/2021

Last edited date: 04/08/2022

Submitted to PROSPERO date: 04/08/2022

PROSPERO registration ID: blinded for review

Registered on RRPSPERO date: 15/08/2022

PROSPERO registration number: blinded for review

**Review title**

Radiomics in giant cell tumor of bone (GCTB): a systematic review

**Anticipated or actual stat date**

15/07/2022

**Anticipated or actual completion date**

31/12/2022

**Review question**

The application of radiomics in giant cell tumor of bone (GCTB) for diagnosis, prediction or prognosis.

**Searches**

Primary publications concerning radiomics or image texture analysis of CT, MRI, PET/CT or PET/MR in patients with GCTB will be included in this review. Electronic databases including PubMed, Embase, Web of Science, China National Knowledge Infrastructure, and Wanfang Data will be searched. Literature search strategies will be developed using medical subject headings (MeSH) and derived words, including radiomics, textural analysis, CT, MR, PET, GCTB, etc. No restriction will be made regarding publication period. The search strategy will include only terms relating to the review question. Publications must be available in English, Japanese, Chinese, German or French.

**Condition or domain being studied**

Giant cell tumor of bone (GCTB) is one of the most common intermediate bone tumors, which occurs in young adults 20– 40 years old with a high recurrence rate (20%–50%) and a potential for aggressive behavior. Imaging examinations play an important role in diagnosis and differential diagnosis, therapy response evaluation as well as prognosis prediction of GCTB. However, radiological practice relies mainly on the subjective interpretation of imaging data by an expert radiologist and therefore is dependent on reader experience. Quantitative, reader independent analysis, i. e. radiomics model or texture analysis, may supplement expert opinion and improve diagnostic, predictive and prognostic accuracy. This systematic review will study the application of radiomics model or texture analysis in human patients with GCTB.

**Participants/population**

Participants’ inclusion criteria:

1) patients with histologically confirmed GCTB;

2) patients had undergone at least one pre- treatment pre- or post-treatment CT, MRI, PET/CT or PET/MR;

3) a radiomics model or texture analysis for stratification of tumor, prediction of response to therapy or prognosis of patients was established.

Participants’ exclusion criteria:

1) not human patients, e. g. cell line, xenotransplant;

2) not GCTB, e. g. osteosarcoma, Ewing sarcoma;

3) no performed imaging procedure;

4) no radiomics model established or texture analysis performed.

**Intervention(s), exposure(s)**

Patients with GCTB underwent at least one pre- treatment pre- or post-treatment CT, MRI, PET/CT or PET/MR with a radiomics model or texture analysis performed based on these imaging data.

**Comparator(s)/control**

Standard-of-care imaging.

**Types of study to be included**

Studies describing radiomics model or texture analysis of CT, MRI, PET/CT or PET/MR in patients with GCTB for diagnosis purpose, stratification of tumor, prediction of response to therapy or prognosis will be included in this review. Studies must be with full-text available and sufficient information for assessing the methodological quality.

Study inclusion criteria:

1) studies are reported in English, Japanese, Chinese, German or French with institutional full-text availability;

2) the cohort consists of patients with histologically confirmed GCTB;

3) patients had undergone at least one pre- treatment pre- or post-treatment CT, MRI, PET/CT or PET/MR;

4) a radiomics model or texture analysis for stratification of tumor, prediction of response to therapy or prognosis of patients was established.

Study exclusion criteria:

1) duplicate studies;

2) reviews, technical reports, letters to editors, comments to published studies, conference proceedings, case reports, brief communications and articles with insufficient information for assessing the methodological quality;

3) studies are reported other than English, Japanese, Chinese, German or French;

4) not human, not GCTB, not radiomics or texture analysis studies.

**Main outcome(s)**

The characteristics of included study will be summarized. The methodological quality, reporting quality, image pre-processing steps, and risk of bias and concern on application, will be assessed.

**Measures of effect**

The studies will be assessed by Radiomics Quality Score (RQS), Transparent Reporting of a multivariable prediction model for Individual Prognosis Or Diagnosis (TRIPOD) statement, Checklist for Artificial Intelligence in Medical Imaging (CLAIM), and modified Quality Assessment of Diagnostic Accuracy Studies (QUADAS-2) tool.

**Additional outcome(s)**

If a sufficient number of studies attempts to answer a similar question, a meta-analysis may be performed to present the performance of those radiomics models.

**Measures of effect**

Measures will be made during the data analysis phase.

**Data extraction (selection and coding)**

A data collection tool will be established based on similar reviews and then trialed on two randomly chosen studies, which fulfilled all the inclusion criteria. These shall be used to train reviewers to appropriately apply the data extraction tool.

**Risk of bias (quality) assessment**

The risk of bias and quality of studies will be assessed by Radiomics Quality Score (RQS), Transparent Reporting of a multivariable prediction model for Individual Prognosis Or Diagnosis (TRIPOD) statement, Checklist for Artificial Intelligence in Medical Imaging (CLAIM), and modified Quality Assessment of Diagnostic Accuracy Studies (QUADAS-2) tool.

**Strategy for data synthesis**

A narrative synthesis will be provided with information presented in the text and/or tables to summarize and explain the characteristics and findings of the included studies. A quantitative synthesis will be done if the included studies are sufficiently homogenous. All analysis will be based on aggregate data.

**Analysis of subgroups or subsets**

If a sufficiently homogeneous subset of studies analyzed a single outcome parameter, e. g. differential diagnosis models based on radiomics or texture analysis, a meta-analysis of this subgroup may be attempted.

**Type and method of review**

Diagnostic; Prognostic; Systematic review; Cancer; Musculoskeletal

**Dissemination plans**

We planned to publish the systematic review via peer-reviewed journals.

**Keywords**

Giant cell tumor of bone, GCTB, radiomics

**Conflicts of interest**

The authors declare that they have no competing interests.

**Supplementary Note S2 Search strategy and study selection**

**1. Search strategy**

**1.1 PubMed Search Strategy**

Avalable via https://pubmed.ncbi.nlm.nih.gov

Preliminary search date: 21 Jun 2021

Articles retrieved: 13

Formal search date: 31 Jul 2022

Articles retrieved: 19

Search string:

(GCTB OR osteoclastoma OR 'giant cell' OR 'Giant Cell Tumor of Bone'[Mesh]) AND ('magnetic resonance imaging'[Mesh] OR magnetic resonance imaging OR magnetic resonance OR MRI OR MR OR 'tomography, x-ray computed'[Mesh] OR computed tomography OR CT OR 'positron-emission tomography'[Mesh] OR positron emission tomography OR PET) AND (textural*[Title/Abstract] OR texture*[Title/Abstract] OR radiomics*[Title/Abstract] OR radiomic*[Title/Abstract] OR histogram*[Title/Abstract])

**1.2 Embase Search Strategy**

Available via www.embase.com

Preliminary search date: 21 Jun 2021

Articles retrieved: 3

Formal search date: 31 Jul 2022

Articles retrieved: 5

Search string:

('osteoclastoma'/exp OR 'osteoclastoma' OR 'osteoclastoma':ti,ab,kw OR 'gaint cell' OR 'gaint cell':ti,ab,kw OR 'gaint cell tumor of bone' OR 'gaint cell tumor of bone':ti,ab,kw OR 'gctb' OR 'gctb':ti,ab,kw) AND ('radiomic':ti,ab,kw OR 'radiomics'/exp OR 'radiomics' OR 'radiomics':ti,ab,kw OR 'textural':ti,ab,kw OR 'texture'/exp OR 'texture' OR 'texture':ti,ab,kw OR 'histogram'/exp OR 'histogram' OR 'histogram':ti,ab,kw) AND ('magnetic resonance imaging'/exp OR 'magnetic resonance imaging' OR 'magnetic resonance imaging':ti,ab,kw OR 'magnetic resonance':ti,ab,kw OR mr:ti,ab,kw OR mri:ti,ab,kw OR 'computed tomography'/exp OR 'computed tomography' OR 'computed tomography':ti,ab,kw OR ct:ti,ab,kw OR 'positron emission tomography'/exp OR 'positron emission tomography' OR 'positron emission tomography':ti,ab,kw OR pet:ti,ab,kw)

**1.3 Web of Science Search Strategy**

Available via apps.webofknowledge.com

Preliminary search date: 21 Jun 2021

Articles retrieved: 35

Formal search date: 31 Jul 2022

Articles retrieved: 45

Search string:

(TS=(giant cell) OR TS=(osteoblastoma) OR TS=(giant cell tumor of bone)) AND (TS=(radiomic*) OR TS=(radiomics*) OR TS=(textural*) OR TS=(texture*) OR TS=(histogram*)) AND (TS=(magnetic resonance imaging) OR TS=(magnetic resonance) OR TS=(MRI) OR TS=(MR) OR TS=(computed tomography) OR TS=(CT) OR TS=( positron emission tomography) OR TS=(PET))

**1.4 China National Knowledge Infrastructure Search Strategy**

Available via http://www.cnki.net

Preliminary search date: 30 Sep 2021

Articles retrieved: 5

Formal search date: 31 Jul 2022

Articles retrieved: 9

Search string:

TKA="巨细胞瘤" AND (TKA="影像组学" OR TKA="直方图" OR TKA= "纹理")

English translation:

giant cell tumor AND (radiomics OR histogram OR texture)

**1.5 Wanfang Data Search Strategy**

Available via https://www.wanfangdata.com.cn

Preliminary search date: 30 Sep 2021

Articles retrieved: 5

Formal search date: 31 Jul 2022

Articles retrieved: 7

Search string:

"巨细胞瘤" AND ("影像组学" OR "直方图" OR "纹理")

English translation:

giant cell tumor AND (radiomics OR histogram OR texture)

This study search strategy has been tested in a pilot search to confirm its feasibility on 21 Jun 2021. The formal study search was performed on 31 Jul 2022.

**2. Study selection**

**2.1 Study for systematic review**

Study inclusion criteria:

1) studies are reported in English, Japanese, Chinese, German or French with institutional full-text availability;

2) the cohort consists of patients with histologically confirmed GCTB;

3) patients had undergone at least one pre- treatment pre- or post-treatment CT, MRI, PET/CT or PET/MR;

4) a radiomics model or texture analysis for stratification of tumor, prediction of response to therapy or prognosis of patients was established.

Study exclusion criteria:

1) duplicate studies;

2) reviews, technical reports, letters to editors, comments to published studies, conference proceedings, case reports, brief communications and articles with insufficient information for assessing the methodological quality;

3) studies are reported other than English, Japanese, Chinese, German or French;

4) not human, not GCTB, not radiomics or texture analysis studies.

Contact with the authors was sought if the full-text version was not accessible otherwise. The reference lists of included studies and relevant reviews identified through the search were screened for additional, potentially eligible articles. Two reviewers with 4- and 5-year-experience in radiology and radiomics research, respectively, screened and selected studies independently. One of these two reviewers can read articles in English, Chinese, Japanese, German and French. In case of disagreements, a third reviewer with 30-year-experience in musculoskeletal radiology would be consulted.

**2.2 Study for meta-analysis**

As predetermined in the review protocol, if a sufficient number of studies attempts to answer a similar question, a meta-analysis could be performed. In current study, the meta-analysis was not conducted, because there was not a sufficient number of studies attempts to answer a similar question with homogeneity. The whole review group discussed this issue and agreed to give up the meta-analysis. We planned to meta-analyze the data if a sufficient number of studies is available.

**Supplementary Note S3 Consensus reached during data extraction and quality assessment**

**1. RQS, TRIPOD and CLAIM**

The RQS consists of 16 items concerning crucial aspects of radiomics studies, to assess their methodological quality. The reviewers performed RQS evaluation according to six key domains as previous reported. The TRIPOD checklist, consisting of 37 items in 22 criteria, was applied to determine the reporting completeness of the included prediction models. Since the TRIPOD checklist was originally produced for the clinical prediction model, it was partially modified for application in radiomics studies. The CLAIM is developed after the Standards for Reporting of Diagnostic Accuracy Study (STARD) guideline, and has been demonstrated as a useful tool to improve design and reporting of deep learning researches. This checklist is designed for clear, transparent and reproducible scientific communication about the application of AI in medical imaging. The CLAIM includes forty-two items in seven topics that should be viewed as a best practice to guide presentation of AI research. The CLAIM has seldomly been employed for quality assessment of radiomics studies. However, we assumed that CLAIM is suitable for radiomics studies evaluation, as radiomics is a subset of AI application in medical imaging.

The previous reviews have well discussed the use of this tool and established a shared point of view, and our review group almost agree with the previous reviews. Therefore, only a limited number of topics needs further discussion. Two reviewers who with 4- and 5-year-experience in radiology and radiomics research, respectively, discussed with a third reviewer with 30-year-experience in musculoskeletal radiology, and made a consensus on additional topics of RQS, TRIPOD, and CLAIM considering the characteristics of current review.

The following topics reached a consensus:

**(1) Gold standard (RQS #13), Outcome - clearly define the outcome, including how and when assessed (TRIPOD #6a), and Benchmark of performance (CLAIM #35b):** These items were considered different in three checklists. The TRIPOD #6a and CLAIM #35b both referred to the method of the real “gold standard” for the outcome, e. g., pathohistological assessment of diagnostic models, or follow-up for prognostic model. However, the so-called “gold standard” (RQS #13) referred to the best method to evaluate the outcome before the final results was available, e. g. radiologists’ assessment before the pathohistological diagnosis, or scoring systems for prediction of prognosis in patients, such as TMN system in multiple cancers or Ennkecking staging in bone sarcomas, before the follow-up.

**(2) Validation (RQS #12), Model type (TRIPOD), and Validation or testing on external data (CLAIM #32):** The validation was the key issue in the radiomics process. In the RQS, if cross-validation or nested cross-validation was performed only within the training set, it was considered missing validation and scored -5 points, as previously described. If validation was performed on a dataset from the same institution, it scored +2 points. If the validation was based on a dataset from another institute, it scored +3 points. However, in the TRIPOD, the external validation is not classified in detail. The separation method (random or non-random) was more important in TRIPOD. In the CLAIM, the datasets were divided into training, validation, and testing datasets. The validation datasets in RQS and TRIPOD were equal to the testing datasets in CLAIM.

**(3) Provided reproducible model description (CLAIM #22a), and Robustness or sensitivity analysis (CLAIM #30):** These two items were different. The CLAIM #22a item needs the article to provide a complete and detailed structure of the model, including inputs, outputs, and all intermediate layers, in sufficient detail that another investigator could exactly re-construct the network. We considered this item as complete if all three elements, model type (e.g., logistic regression, Cox proportional hazards model), feature selection procedure to control overfitting, and methods of internal validation (cross-validation, bootstrap sample), were included. A regularization or penalization method such as the least absolute shrinkage and selection operator (LASSO) was considered as both a feature selection procedure and internal validation, as it contains 10-fold cross-validation as a default setting. On the other hand, the CLAIM #30 item includes the various assumptions or initial conditions which potentially influenced on the robustness or sensitivity of the model.

**(4) Multiple segmentations (RQS #2), Measurement of inter- and intra-rater variability (CLIAM #18):** These two items were similar. In the RQS, the segmentation by different physicians/algorithms/software, perturbing segmentations by (random) noise, segmentation at different breathing cycles. Analyze feature robustness to segmentation variabilities. In the CLAIM, the item referred to the methods to measure inter- and intra- rater variability, and any steps taken to reduce or mitigate this variability and/or resolve discrepancies. For radiomics studies, this mainly refers to variability between readers, as well we other measurements, such as image interoperations.

**(5) Level at which partitions are disjoint (e.g., image, study, patient, institution) (CLAIM #21):** The item referred to the level at which the partitions are disjoint. Sets of medical images generally should be disjoint at the patient level or higher so that images of the same patient do not appear in each partition. Although this item was seldomly described in the manuscript, we can find it out through the presentation of the results.

**2. Risk of Bias and Concern on Application Assessment according to QUADAS-2 Tool**

The QUADAS-2 tool was developed for the risk of bias and concern of application assessment. Two reviewers who with 4- and 5-year-experience in radiology and radiomics research, respectively, discussed with a third reviewer with 30-year-experience in 30-year-experience in musculoskeletal radiology, and made a consensus on additional topics of QUADAS-2 considering the characteristics of current review. The previous reviews have well discussed the use of this tool and established a shared point of view, and our review group almost agree with the previous reviews. Therefore, we applied the signal questions to guide our review.

**Supplementary Note S4 Data synthesis and analysis methods**

**1. Statistical Analysis**

The SPSS software version 26.0 was used for statistical analysis. A two-tailed *p*-value < 0.05 was recognized as statistical significance, unless otherwise specified. 16 items of the RQS were scored. The RQS score and percentage of the ideal score were described as score and percentage of score to ideal score for each item, respectively. A total of 37 items and subitems on the TRIPOD checklist was scored. During the calculation of TRIPOD, the “if done” or “if relevant” items (5c, 11, and 14b) and validation items (10c, 10e, 12, 13, 17, and 19a) were excluded from both the denominator and numerator. A total of 53 items and subitems of CLAIM was scored. During the calculation of CLAIM, the “if applicable” item (27) was excluded from both the denominator and numerator. In the cases where a score of one point per item was obtained, the study was considered to have basic adherence to each item of the RQS rating, TRIPOD checklist and CLAIM. For example, if the item of validation in RQS obtained 2 to 5 points, it was considered as basic adherent, while it was regarded as without basic adherence when it was rated as -5 points. The adherence rate of RQS rating, TRIPOD checklist and CLAIM were calculated as proportion of the number of articles with basic adherence to number of total articles. The result of QUADAS-2 risk of bias and application concern assessment was summarized as proportions of high risk, low risk and unclear.

Subgroup analysis was not performed due to insufficient number of studies. The correlation among between RQS, TRIPOD, sample size, impact factor, and other potential factors was not conducted also due to insufficient number of studies. We planned to investigate the potential factors that influence on the study quality ratings when a sufficient number of studies is available.

**2. Meta-analysis**

As predetermined in the review protocol, if a sufficient number of studies attempts to answer a similar question, a meta-analysis could be performed. In current study, the meta-analysis was not conducted, because there was not a sufficient number of studies attempts to answer a similar question with homogeneity. The whole review group discussed this issue and agreed to give up the meta-analysis. We planned to meta-analyze the data when a sufficient number of studies is available.

**3. Clinical value and Level of Evidence**

To assess the gap from clinical application, the evidence rating process is recommended. However, the rating process assesses the pieces of evidence supporting clinical values of radiomics models based on results of meta-analysis. Therefore, the process is currently not available for our study. We planned to rate the evidence level of radiomics in GCTB, when meta-analysis is available.

**4. Feature-level analysis**

Most of the previous systematic reviews focused on the model performance metrics, and usually conclude that the imaging biomarkers are “promising”. However, biomarker research is commonly haphazard, inconsistent, and underpowered, the “promising” results of these studies may be due to methodological error rather than intrinsic ability.

Our study, therefore, introduced a new radiomics feature-level analysis for systematic reviews of radiomics studies. We planned to identify radiomics features that appears repeatably in multiple studies concerning on the same or similar clinical questions, and then perform a qualitative or quantitative evaluation. If we assume genuinely promising biomarkers will appear in multiple studies, these may then be identified via systematic review. This process can identify those appearing “promising” due to intrinsic ability rather than methodological errors, i. e., it can reduce the likelihood that radiomic features are selected by statistical chance or may be attributed to the nature of the data sample used for model development. With sufficient primary data, their estimates can be meta-analyzed to obtain a signal of whether a predictor has genuine promise.

We assumed that only radiomics features that appears repeatably in multiple studies are considered to be with biological validity. For example, a radiomic signature that is related to survival outcomes may potentially reflect a tissue phenotype associated with a specific biology. Therefore, this process can serve as a preliminary study for determination of the histopathological meanings of radiomics features, and avoid deficiencies in current studies and checklist that mainly focus on the technical and clinical validity.

In current study, we found that there is a limited number of features appears repeatably in the radiomics models included studies. Therefore, a quantitative analysis was not performed. We qualitatively summarized the group of selected features in radiomics models answering different clinical questions to show the status quo of GCTB radiomics. We planned to perform quantitative analysis when a sufficient number of studies is available.

However, we believe the attempt of feature-level analysis of radiomics study is necessary for promoting the translation of radiomics as an imaging biomarker into clinical practice. We also planned to performed such an analysis in other diseases with sufficient number of studies (e. g. glioma, lung cancer, or prostate cancer), to present the usefulness of this process.

**Supplementary Table S1 Data extraction instrument**

| **Field** | **Item** |
| --- | --- |
| Bibliographical Information | The Title of The Study |
|  | The First Authorship of The Study |
|  | Published Year |
|  | Published Journal |
|  | Impact Factor of Published Journal |
|  | Published Volume |
|  | Published Issue |
|  | Published Page |
|  | Country |
|  | Study ID, determined by First Author + Year, + Journal if needed |
| Study Characteristics | Study Design |
|  | Patient Condition |
|  | Patient Gender |
|  | Patient Age |
|  | Imaging Modality |
|  | Predictor |
|  | Outcome |
|  | Reference Standard |
|  | Data Splitting |
| Radiomics Considerations | ROI Segmentation |
|  | Radiomics Feature Extraction Details |
|  | Radiomics Feature Reduction Details |
|  | Radiomics Feature Selection Details |
|  | Selector |
| Model Metrics | Sample Size |
|  | Number of Events (True Positive, False Positive, False Negative, True Negative) |
|  | Sensitivity |
|  | Specificity |
|  | Accuracy |
|  | Positive Predictive Value (PPV) |
|  | Negative Predictive Value (NPV) |
|  | Positive Likelihood Ratio (PLR) |
|  | Negative Likelihood Ratio (NLR) |
|  | Diagnostic Odds Ratio (DOR) |

Note: The radiomics feature-level data were specially emphasized during the data extraction process.

**Supplementary Table S2 RQS elements according to six key domains**

| **Domain** | **RQS#** | **RQS scoring item** | **Points and Interpretation** |
| --- | --- | --- | --- |
| **Domain 1:** Protocol quality and stability in image and segmentation (0 to 5) | 1 | **Image protocol quality** - well-documented image protocols (for example, contrast, slice thickness, energy, etc.) and/or usage of public image protocols allow reproducibility/replicability | + 1 if protocols are well-documented  + 1 if public protocol is used |
|  | 2 | **Multiple segmentations** - possible actions are: segmentation by different physicians/algorithms/software, perturbing segmentations by (random) noise, segmentation at different breathing cycles. Analyse feature robustness to segmentation variabilities | + 1 if segmented multiple times (different physicians, algorithms, or perturbation of regions of interest) |
|  | 3 | **Phantom study on all scanners** - detect inter-scanner differences and vendor-dependent features. Analyse feature robustness to these sources of variability | + 1 if texture phantoms were used for feature robustness assessment |
|  | 4 | **Imaging at multiple time points** - collect images of individuals at additional time points. Analyse feature robustness to temporal variabilities (for example, organ movement, organ expansion/ shrinkage) | + 1 multiple time points for feature robustness assessment |
| **Domain 2:** Feature selection and validation (-8 to 8) | 5 | **Feature reduction or adjustment for multiple testing** - decreases the risk of overfitting. Overfitting is inevitable if the number of features exceeds the number of samples. Consider feature robustness when selecting features | - 3 if neither measure is implemented  + 3 if either measure is implemented |
|  | 12 | **Validation** - the validation is performed without retraining and without adaptation of the cut-off value, provides crucial information with regard to credible clinical performance | − 5 if validation is missing  + 2 if validation is based on a dataset from the same institute/  + 3 if validation is based on a dataset from another institute/  + 4 if validation is based on two datasets from two distinct institutes/  +4 if the study validates a previously published signature/  +5 if validation is based on three or more datasets from distinct institutes  *Datasets should be of comparable size and should have at least 10 events per model feature |
| **Domain 3:** Biologic/clinical validation and utility (0 to 6) | 6 | **Multivariable analysis with non-radiomics features** (for example, EGFR mutation) - is expected to provide a more holistic model. Permits correlating/inferencing between radiomics and non-radiomics features | + 1 if multivariable analysis with non-radiomics features |
|  | 7 | **Detect and discuss biological correlates** - demonstration of phenotypic differences (possibly associated with underlying gene–protein expression patterns) deepens understanding of radiomics and biology | + 1 if present |
|  | 13 | **Comparison to gold standard** - assess the extent to which the model agrees with/is superior to the current ‘gold standard’ method (for example, TNM-staging for survival prediction). This comparison shows the added value of radiomics | + 2 for comparison to gold standard |
|  | 14 | **Potential clinical utility** - report on the current and potential application of the model in a clinical setting (for example, decision curve analysis) | + 2 for reporting potential clinical utility |
| **Domain 4:** Model performance index (0 to 5) | 8 | **Cut-off analyses** - determine risk groups by either the median, a previously published cut-off or report a continuous risk variable. Reduces the risk of reporting overly optimistic results | + 1 if cutoff either pre-defined or at median or continuous risk variable reported |
|  | 9 | **Discrimination statistics** - report discrimination statistics (for example, C-statistic, ROC curve, AUC) and their statistical significance (for example, p-values, confidence intervals). One can also apply resampling method (for example, bootstrapping, cross-validation) | + 1 if a discrimination statistic and its statistical significance are reported  + 1 if a resampling method technique is also applied |
|  | 10 | **Calibration statistics** - report calibration statistics (for example, Calibration-in-the-large/slope, calibration plots) and their statistical significance (for example, *P*-values, confidence intervals). One can also apply resampling method (for example, bootstrapping, cross-validation) | + 1 if a calibration statistic and its statistical significance are reported  + 1 if a resampling method technique is also applied |
| **Domain 5:** High level of evidence (0 to 8) | 11 | **Prospective study registered in a trial database** - provides the highest level of evidence supporting the clinical validity and usefulness of the radiomics biomarker | + 7 for prospective validation of a radiomics signature in an appropriate trial |
|  | 15 | **Cost-effectiveness analysis** - report on the cost-effectiveness of the clinical application (for example, QALYs generated) | + 1 for cost-effectiveness analysis |
| **Domain 6:** Open science and data (0 to 4) | 16 | **Open science and data** - make code and data publicly available. Open science facilitates knowledge transfer and reproducibility of the study | + 1 if scans are open source  + 1 if region of interest segmentations are open source  + 1 if code or software is open source  + 1 if radiomics features are calculated on a set of representative ROIs and the calculated features and representative ROIs are open source |
|  | Total points (36 = 100%) | | |

Note: RQS = Radiomics Quality Score.

Extracted from Lambin P, Leijenaar RTH, Deist TM, et al. Radiomics: the bridge between medical imaging and personalized medicine. Nat Rev Clin Oncol. 2017;14(12):749-762.

**Supplementary Table S3 TRIPOD reporting completeness checklist**

| **Section** | **TRIPOD#** | **Item** | **Explanation** | **Values** |
| --- | --- | --- | --- | --- |
| **Title and Abstract** | 1 | **Title** - identify developing/validating a model, target population, and the outcome | #1: considered as complete if all elements of the type of study (development, validation, incremental value or combination), the target population, and outcome are included. | 0. Not documented  1. Complete |
|  | 2 | **Abstract** - provide a summary of objectives, study design, setting, participants, sample size, predictors, outcome, statistical analysis, results, and conclusions | #2 and #3b: considered as complete if ‘development’ and/or ‘validation’ is explicitly written. Synonyms instead of development such as ‘establish’, ‘build’, ‘investigate’, and ‘evaluate’ were not considered as complete. | 0. Not complete  1. Complete |
| **Introduction** | 3a | **Background** - Explain the medical context and rationale for developing/validating the model | #3a: considered as complete if at least a simple sentence was provided to introduce the medical context and rationale for developing/validating the model. | 0. Not complete  1. Complete |
|  | 3b | **Objective** - Specify the objectives, including whether the study describes the development/validation of the model or both. | #2 and #3b: considered as complete if ‘development’ and/or ‘validation’ is explicitly written. Synonyms instead of development such as ‘establish’, ‘build’, ‘investigate’, and ‘evaluate’ were not considered as complete | 0. Not complete  1. Complete |
| **Methods** | 4a | **Source of data** - describe the study design or source of data (randomized trial, cohort, or registry data) | #4a: whether the study was conducted in a randomized controlled trial, cohort, or registry with a consecutive, random, or convenience series. A study was considered as complete when the terms ‘retrospective’ or ‘prospective’ were mentioned. | 0. Not documented  R. Retrospective  P. Prospective  RP. Both retrospective and prospective |
|  | 4b | **Source of data** - specify the key dates | #4b: provide the name of open-source data, or declaim that the study was performed based on institutional dataset with a specific inclusion period. | 0. Not documented  L. Local data collection  P. Public data  LP. Both local and public data |
|  | 5a | **Participants** - specify key elements of the study setting including number and location of centers | #5a: number and location of centers should be declared in multicenter studies; monocenter study should state the location of that the study performed. | 0. Not documented  SC. Single-center data  MC. Multi-center data |
|  | 5b | **Participants** - describe eligibility criteria for participants (inclusion and exclusion criteria) | #5b: considered as complete if a structured criterion of inclusion and exclusion were provided; only disease name was not considered as complete. | 0. Not documented  1. Documented |
|  | 5c | **Participants** - give details of treatment received, *if relevant* | #5c: treatments are relevant in prognostic studies as they modify outcomes and relevant information should be reported. | 0. Not documented  1. Documented |
|  | 6a | **Outcome** - clearly define the outcome, including how and when assessed | #6a: the method of assessment, e.g., histology and experience of pathologists; follow-up, frequency and modality; or expert’s opinion and experience of experts. | 0. Not defined  1. Defined either explicitly or by reference to a  Common Data Element |
|  | 6b | **Outcome** - report any actions to blind assessment of the outcome | #6b: describe weather the outcome is ideally assessed while blinded to information about the predictors. | 0. Not documented  1. Documented |
|  | 7a | **Predictors** - clearly define all predictors, including how and when assessed | #7a: the radiomics studies involve quantitative feature extraction through an automated process; thus, the element ‘when’ was ignored. | 0. Not documented  1. Documented |
|  | 7b | **Predictors** - report any actions to blind assessment of predictors for the outcome and other predictors | #7b: if radiomics studies were based on regions-of-interest and the blindness of readers to the reference standard was considered, they were recorded as complete. If ‘blind’ or ‘unaware of’ the reference standard was not explicitly written, it was considered as incomplete. Automatic segmentation was considered as complete. | 0. Not documented  1. Documented |
|  | 8 | **Sample size** - explain how the study size was arrived at | #8: considered as complete if the database, software or method, and results were described. | 0. Not documented  1. Documented |
|  | 9 | **Missing data** - describe how missing data were handled with details of any imputation method | #9: considered as complete if the imputation method was described when there is missing data, or how to excluded the insufficient data when imputation was not performed | 0. Not documented  E. Missing data excluded from analysis  I. Missing data included in analysis |
|  | 10a | **Statistical analysis methods** - describe how predictors were handled | #10a: considered as complete if the statistical analysis method (e.g., t test, chi-square test) were included, and suitable for the variable type. | 0. Not documented  1. Documented |
|  | 10b | **Statistical analysis methods** - specify type of model, all model-building procedures (any predictor selection), and method for internal validation | #10b: considered as complete if all three elements, model type (e.g., logistic regression, Cox proportional hazards model), feature selection procedure to control overfitting, and methods of internal validation (cross-validation, bootstrap sample), were included. A regularization or penalization method such as the least absolute shrinkage and selection operator (LASSO) was considered as both a feature selection procedure and internal validation, as it contains 10-fold cross-validation as a default setting. | 0. Not documented  1. Documented |
|  | 10d | **Statistical analysis methods** - specify all measures used to assess model performance and if relevant, to compare multiple models (discrimination or calibration) | #10d: the article was considered as complete if both the discrimination and calibration index were written | 0. Not documented  1. Documented |
|  | 11 | **Risk groups** - provide details on how risk groups were created, if done | #11: considered as complete if the cutoffs were provided, e.g., disease stage, predictive absolute incidence, or risk rate. | 0. Not documented  1. Documented |
| **Results** | 13a | **Participants** - describe the flow of participants, including the number of participants with and without the outcome. A diagram may be helpful. | #13a: considered as complete if a diagram or text description with the numbers of screened patients, excluded patients and included patients was provided. | 0. Not documented  1. Documented |
|  | 13b | **Participants** - describe the characteristics of the participants, including the number of participants with missing data for predictors and outcome | #13b: considered as complete if a table or text description was provided. | 0. Not documented  1. Documented |
|  | 14a | **Model development** - specify the number of participants and outcome events in each analysis | #14a: considered as complete if a table or text description was provided. | 0. Not documented  1. Documented |
|  | 14b | **Model development** - report the unadjusted association between each candidate predictor and outcome, *if done* | #14b: considered as complete if the metrics and their confidence interval were provided. | 0. Not documented  1. Documented |
|  | 15a | **Model specification** - present the full prediction model to allow predictions for individuals (regression coefficients, intercept) | #10c, #15a, and #15b: these items determine if an article describes how the obtained model predicted the outcome probabilities for an individual. If the articles described this in the methods (item 10c) and contained a full prediction model including all regression coefficients and the intercept or baseline hazard for a particular time point, they were considered as complete for item 15a. If the study contained explicit formula or a nomogram, the study was considered as complete for item 15b. | 0. Not documented  1. Documented |
|  | 15b | **Model specification** - explain how to the use the prediction model (nomogram, calculator, etc) | #10c, #15a, and #15b: these items determine if an article describes how the obtained model predicted the outcome probabilities for an individual. If the articles described this in the methods (item 10c) and contained a full prediction model including all regression coefficients and the intercept or baseline hazard for a particular time point, they were considered as complete for item 15a. If the study contained explicit formula or a nomogram, the study was considered as complete for item 15b. | 0. Not documented  1. Documented |
|  | 16 | **Model performance** - report performance measures (with confidence intervals) for the prediction model | #16: considered as complete if the metrics (at least the discrimination outcome) and their confidence interval were provided. | 0. Diagnostic performance reported without measure of precision  1. Diagnostic performance reported with confidence interval or standard error |
| **Discussion** | 18 | **Limitations** - Discuss any limitations of the study | #18: considered as complete if there was a limitation paragraph, usually the paragraph before the conclusion. | 0. Not discussed  1. Discussed |
|  | 19b | **Interpretation** - Give an overall interpretation of the results | #19b: considered as complete if there was an interpretation of results paragraph, usually the paragraph of discussion. | 0. Not documented  1. Documented |
|  | 20 | **Implications** - Discuss the potential clinical use of the model and implications for future research | #20: considered as complete if there was text description or decision curve analysis. This is different from ‘clinical validity’ in RQS criterion 14, that the decision curve analysis was necessary. | 0. Not discussed  1. Discussed |
| **Validation (types 2a, 2b, 3, and 4)** | 10c | **Statistical analysis methods** - describe how the predictions were calculated | #10c, #15a, and #15b: these items determine if an article describes how the obtained model predicted the outcome probabilities for an individual. If the articles described this in the methods (item 10c) and contained a full prediction model including all regression coefficients and the intercept or baseline hazard for a particular time point, they were considered as complete for item 15a. If the study contained explicit formula or a nomogram, the study was considered as complete for item 15b. | 0. Not documented  1. Documented |
|  | 10e | **Statistical analysis methods** - describe any model updating (recalibration), *if done* | #10e and #17: If an article describes methods to adjust (recalibrate) or update a previously developed prediction model, the article is scored. This is different from ‘comparison with gold standard’ in RQS criterion 13, in that it requires recalibration of regression coefficients and hazard ratios in the pre-existing model, and was scored if it was completely reported. | 0. Not documented  1. Documented  n/a. Not updating |
|  | 12 | **Development vs. validation** - Identify any differences from the development data in setting, eligibility criteria, outcome, and predictors | #12 and #13c: considered as complete if a table comparing developing and testing dataset or text description was provided. | 0. Not documented  1. Documented |
|  | 13c | **Participants (for validation)** - show a comparison with the development data of the distribution of important variables | #12 and #13c: considered as complete if a table comparing developing and testing dataset or text description was provided. | 0. Not documented  1. Documented |
|  | 17 | **Model updating** - report the results from any model updating, *if done* | #10e and #17: If an article describes methods to adjust (recalibrate) or update a previously developed prediction model, the article is scored. This is different from ‘comparison with gold standard’ in RQS item 13, in that it requires recalibration of regression coefficients and hazard ratios in the pre-existing model, and was scored if it was completely reported. | 0. Not documented  1. Documented  n/a. Not updating |
|  | 19a | **Interpretation** **(for validation)** - discuss the results with reference to performance in the development data and any other validation data | #19a: considered as complete if there was a paragraph that discuss the influence of difference between development and validation data on the model performance. The performance of the model in the validation study should be discussed and placed in context to the model performance in the original development study and with any other existing validation studies of that model. One should highlight the main results, as well as any biases that may have affected the comparison. When the validation study shows a different (usually poorer) performance, reasons should be discussed to enhance interpretation. | 0. Not documented  1. Documented |
| **Other Information** | 21 | **Supplementary information** - provide information about the availability of supplementary resources, such as study protocol, Web calculator, and data sets | #21: considered as complete if the study provided supplementary materials and/or links for online resources, or declared that all data were provided in the manuscript. | 0. Not documented  1. Documented |
|  | 22 | **Funding** - give the source of funding and the role of the funders for the present study | #22: considered as complete if the source of funding and the role of the funders were both declared. | 0. Not documented  F. Funding source documented  FR. Funding source and role documented  NF. Stated no funding received |

Note: TRIPOD = Transparent Reporting of a multivariable prediction model for Individual Prognosis Or Diagnosis. Extracted from Collins GS, Reitsma JB, Altman DG, Moons KG. Transparent reporting of a multivariable prediction model for individual prognosis or diagnosis (TRIPOD): the TRIPOD statement. Ann Intern Med. 2015;162(1):55-63.

Supplementary Table S4 CLAIM for authors and reviewers

| **Section / Topic** | **CLAIM#** | **Item** | **Explanation** | **Values** |
| --- | --- | --- | --- | --- |
| **TITLE / ABSTRACT** | | | | |
| **Title or abstract** | 1 | Identification as a study of AI methodology | #1: considered as complete if the title and/ or abstract indicates the usage of AI methodology, such as histogram, texture analysis, radiomics, machine learning (or specific machine learning method), or deep learning (or neuron network). | 0. Not specified  1. Specified |
| **Abstract** | 2 | Structured summary of study design, methods, results, and conclusions. | #2: considered as complete if all following items is provided: study design, methods, results, and conclusions. However, the original version recommended to present: (1) Provide an overview of the study population (number of patients or examinations, number of images, age and sex distribution). (2) Indicate if the study is prospective or retrospective, and summarize the statistical analysis that was performed. (3) When presenting the results, be sure to include *P* values for any comparisons. (4) Indicate whether the software, data, and/or resulting model are available publicly. | 0. Not included  1. Included |
| **INTRODUCTION** | | | | |
| **Background** | 3 | Scientific and clinical background, including the intended use and clinical role of the AI approach | #3: considered as complete if at least a simple sentence was provided to introduce the medical context and rationale for developing/validating the model. Address an important clinical, scientific, or operational issue. Describe the study’s rationale, goals, and anticipated impact. Summarize related literature and highlight how the investigation builds upon and differs from that work. Guide readers to understand the context for the study, the underlying science, the assumptions underlying the methodology, and the nuances of the study. | 0. Not provided  1. Provided |
| **Study objectives and hypotheses** | 4a | Study objectives | #4a and #4b: considered as complete if there are two sentences describe the aim/ purpose/ objective of the study (4a), and/ or the hypothesis of the study (4b), respectively. Define clearly the clinical or scientific question to be answered; avoid vague statements or descriptions of a process. Limit the chance of post hoc data dredging by specifying the study’s hypothesis a priori. Identify a compelling problem to address. The study’s objectives and hypothesis will guide sample size calculations and whether the hypothesis will be supported or not. | 0. Not provided  1. Provided |
|  | 4b | Study hypotheses |  | 0. Not documented  1. Documented |
| **METHODS** | | | | |
| **Study Design** | 5 | Prospective or retrospective study | #5: considered as complete if the study indicates whether the study is retrospective or prospective. It is recommended to evaluate predictive models in a prospective setting if possible, but if not, this item is not considered as incomplete. | 0. Not documented  R. Retrospective  P. Prospective |
|  | 6 | Study goal, such as model creation, exploratory study, feasibility study, non-inferiority trial | #6: considered as complete if the following keywords were indicated. (1) Define the study’s goal, such as model creation, exploratory study, feasibility study, or noninferiority trial. For classification systems, state the intended use, such as diagnosis, screening, staging, monitoring, surveillance, prediction, or prognosis. (2) Indicate the proposed role of the AI algorithm relative to other approaches, such as triage, replacement, or add-on. (3) Describe the type of predictive modeling to be performed, the target of predictions, and how it will solve the clinical or scientific question. | 0. Not documented  1. Documented |
| **Data** | 7a | Data source | #7: each subitems are evaluated respectively. #7a: not documented, local data source, public data source, local and public data source. #7b: not documented, single-center data, multi-center data. #7c: not documented, single vendor, multiple vendors. State the source of data and indicate how well the data match the intended use of the model. Describe the targeted application of the predictive model to allow readers to interpret the implications of reported accuracy estimates. Reference any previous studies that used the same dataset and specify how the current study differs. Adhere to ethical guidelines to assure that the study is conducted appropriately; describe the ethics review and informed consent. Provide links to data sources and/or images, if available. Authors are strongly encouraged to deposit data and/or software used for modeling or data analysis in a publicly accessible repository. | 0. Not documented  L. Local data collection  P. Public data  LP. Both local and public data |
|  | 7b | Data collection institutions |  | 0. Not documented  SC. Single-center data  MC. Multi-center data |
|  | 7c | Imaging equipment vendors |  | 0. Not documented  SV. Single vendor  MV. Multiple vendors |
|  | 7d | Image acquisition parameters |  | 0. Not documented  1. Documented |
|  | 7e | Institutional review board approval |  | 0. Not documented  1. Documented |
|  | 7f | Participant consent |  | 0. Not documented  1. Documented |
|  | 8 | Eligibility criteria: how, where, and when potentially eligible participants or studies were identified (e.g., symptoms, results from previous tests, inclusion in registry, patient-care setting, location, dates) | #8: considered as complete if a structured criterion of inclusion and exclusion were provided; only disease name was not considered as complete. Define how, where, and when potentially eligible participants or studies were identified. Specify inclusion and exclusion criteria such as location, dates, patient-care setting, symptoms, results from previous tests, or registry inclusion. Indicate whether a consecutive, random, or convenience series was selected. Specify the number of patients, studies, reports, and/or images. | 0. Not documented  1. Documented |
|  | 9 | Data pre-processing steps | #9: not documented, pre-processing documented (but not complete), reproducible pre-processing method documented (fulfilled the following 5 key points), documented that pre-processing not employed. Describe preprocessing steps fully and in sufficient detail so that other investigators could reproduce them. (1) Specify the use of normalization, resampling of image size, change in bit depth, and/or adjustment of window/level settings. (2) State whether or not the data have been rescaled, threshold-limited (“binarized”), and/or standardized. (3) Specify how the following issues were handled: regional format, manual input, inconsistent data, missing data, wrong data types, file manipulations, and missing anonymization. (4) Define any criteria to remove outliers. (5) Specify the libraries, software (including manufacturer name and location), and version numbers, and all option and configuration settings employed. | 0. Not documented  P. Pre-processing documented  NP. Documented that pre-processing not employed |
|  | 10 | Selection of data subsets, if applicable (for radiomics studies, segmentation is a necessary step) | #10: not documented, image cropping documented, reproducible image cropping method documented. For radiomics studies, segmentation is a necessary step. Describe the tools and parameters used; if done manually, specify the training of the personnel and the criteria they used. Justify how this manual step would be accommodated in the context of the clinical or scientific problem to be solved. | 0. Not documented  C. Image cropping documented  CM. Reproducible image cropping method documented |
|  | 11 | Definitions of data elements, with references to Common Data Elements | #11: considered as complete, if the predictor and outcome variables is defined. Map them to common data elements, if applicable. | 0. Not defined  1. Not defined |
|  | 12 | De-identification methods | #12: not defined, anonymization documented, reproducible anonymization method documented. Describe the methods by which data have been de-identified and how protected health information has been removed. | 0. Not documented  A. Anonymization documented |
|  | 13 | How missing data were handled | #13: missing data handling strategy not documented, missing data excluded from analysis, missing data included in analysis. State clearly how missing data were handled, such as replacing them with approximate or predicted values. Describe the biases that the imputed data might introduce. | 0. Not documented  E. Missing data excluded from analysis  I. Missing data included in analysis |
| **Ground Truth** | 14 | Definition of ground truth reference standard, in sufficient detail to allow replication | #14: Include detailed, specific definitions of the ground truth annotations, ideally referencing common data elements. Provide an atlas of examples to annotators to illustrate subjective grading schemes (e. g., mild/moderate/severe), and make that information available for review. | 0. Not defined  1. Defined either explicitly or by reference to a Common Data Element |
|  | 15a | Rationale for choosing the reference standard (if alternatives exist) | #15: Describe the rationale for the choice of the reference standard and the potential errors, biases, and limitations of that reference standard. | 0. Not documented  1. Documented  n/a = not applicable |
|  | 15b | Definitive ground truth |  | 0. No definitive ground truth  P. Histopathology  DI. Definitive imaging modality  FU. Case follow-up  PFU. Histopathology and case follow-up  PDI. Histopathology and definitive imaging modality |
|  | 16 | Manual image annotation | #16: Specify the number of human annotators and their qualifications. Describe the instructions and training given to annotators; include training materials as a supplement, if possible. Describe whether annotations were done independently and how any discrepancies among annotators were resolved. | 0. Not documented  UR. Radiologist with unspecified expertise  SR. Radiologist with relevant subspecialist expertise  OC. Other clinician  A. Automatic method |
|  | 17 | Image annotation tools and software | #17: Specify the software used for manual, semiautomated, or automated annotation, including the version number. | 0. Not documented  1. Documented |
|  | 18 | Measurement of inter- and intra-rater variability; methods to mitigate variability and/or resolve discrepancies | #18: Describe the methods to measure inter- and intra- rater variability, and any steps taken to reduce or mitigate this variability and/or resolve discrepancies. For radiomics studies, this mainly refers to variability between readers, as well we other measurements, such as image interoperations. | 0. Not documented  V. Variability statistics documented  M. Aggregation method documented  VM. Variability statistics and aggregation method documented |
| **Data Partitions** | 19a | Intended sample size | Describe the sample size and how it was determined. Use traditional power calculation methods, if applicable, to estimate the required sample size to allow for generalizability in a larger population and how many cases are needed to show an effect. | 0. Not documented  1. Documented number of images in dataset |
|  | 19b | Provided power calculation |  | 0. Not documented  1. Documented |
|  | 19c | Distinct study participants |  | 0. Not documented  N. number of study participants |
|  | 20 | How data were assigned to partitions; specify proportions | #20: Specify how the data were assigned into training, validation (“tuning”), and testing partitions; indicate the proportion of data in each partition and justify that selection. Indicate if there are any systematic differences between the data in each partition, and if so, why. | 0. Not documented  1. Documented |
|  | 21 | Level at which partitions are disjoint (e.g., image, study, patient, institution) | #21: Describe the level at which the partitions are disjoint. Sets of medical images generally should be disjoint at the patient level or higher so that images of the same patient do not appear in each partition. | 0. Not documented  1. Documented partition disjunction at patient level |
| **Model** | 22a | Provided reproducible model description | #22: Provide a complete and detailed structure of the model, including inputs, outputs, and all intermediate layers, in sufficient detail that another investigator could exactly re-construct the network. (1) For neural network models, include all details of pooling, normalization, regularization, and activation in the layer descriptions. Model inputs must match the form of the preprocessed data. Model outputs must correspond to the requirements of the stated clinical problem, and for supervised learning should match the form of the ground truth annotations. If a previously published model architecture is employed, cite a reference that meets the preceding standards and fully describe every modification made to the model. (2) For radiomics studies, this item is considered as complete if all three elements, model type (e.g., logistic regression, Cox proportional hazards model), feature selection procedure to control overfitting, and methods of internal validation (cross-validation, bootstrap sample), were included. A regularization or penalization method such as the least absolute shrinkage and selection operator (LASSO) was considered as both a feature selection procedure and internal validation, as it contains 10-fold cross-validation as a default setting. (3) In some cases, it may be more convenient to provide the structure of the model in code as supplemental data. | 0. Not documented  1. Documented |
|  | 22b | Provided source code |  | 0. Not documented  1. Documented |
|  | 23 | Software libraries, frameworks, and packages | #23: Specify the names and version numbers of all software libraries, frameworks, and packages. Avoid detailed description of hardware unless benchmarking computational performance is a focus of the work. | 0. Not documented  S. Documented software  SV. Documented software and version |
|  | 24 | Initialization of model parameters (e.g., randomization, transfer learning) | #24: Considered as complete, if feature reduction and selection is described for radiomics models, and the parameters is determined by internal or external validations. Indicate how the parameters of the model were initialized. Describe the distribution from which random values were drawn for randomly initialized parameters. Specify the source of the starting weights if transfer learning is employed to initialize parameters. When there is a combination of random initialization and transfer learning, make it clear which portions of the model were initialized with which strategies. | 0. Not documented  1. Documented |
| **Training** | 25 | Details of training approach, including data augmentation, hyperparameters, number of models trained | #25: Completely describe all of the training procedures and hyperparameters in sufficient detail that another investigator could exactly duplicate the training process. This process needed to be performed in a training dataset. | 0. Not documented  1. Documented |
|  | 26 | Method of selecting the final model | #26: Describe the method and performance parameters used to select the best-performing model among all the models trained for evaluation against the held-out test set. If more than one model is selected, justify why this is appropriate. | 0. Not documented  1. Documented model selection criterion, specifying k if k-fold cross validation  employed |
|  | 27 | Ensembling techniques, if applicable | #27: If the final algorithm involves an ensemble of models, describe each model comprising the ensemble in complete detail in accordance with the preceding recommendations. Indicate how the outputs of the component models are weighted and/or combined. | 0. Not documented  1. Documented  n/a. Ensembling not employed |
| **Evaluation** | 28 | Metrics of model performance | #28: Describe the metric(s) used to measure the model’s performance and indicate how they address the performance characteristics most important to the clinical or scientific problem. Compare the presented model to previously published models. | 0. Not documented  1. Documented |
|  | 29 | Statistical measures of significance and uncertainty (e.g., confidence intervals) | #29: Indicate the uncertainty of the performance metrics’ values, such as with standard deviation and/or confidence intervals. Compute appropriate tests of statistical significance to compare metrics. Specify the statistical software. | 0. Not documented  1. Documented |
|  | 30 | Robustness or sensitivity analysis | #30: Analyze the robustness or sensitivity of the model to various assumptions or initial conditions. | 0. Not documented  1. Documented |
|  | 31 | Methods for explainability or interpretability (e.g., saliency maps), and how they were validated | #31: If applied, describe the methods that allow one to explain or interpret the model’s results and provide the parameters used to generate them. Describe how any such methods were validated in the current study. | 0. Not documented  1. Documented |
|  | 32 | Validation or testing on external data | #32: Describe the data used to evaluate performance of the completed algorithm. When these data are not drawn from a different data source than the training data, note and justify this limitation. If there are differences in structure of annotations or data between the training set and evaluation set, explain the differences, and describe and justify the approach taken to accommodate the differences. | 0. Not described  I. Employed internal test data  E. Employed external test data |
| **RESULTS** | | | | |
| **Data** | 33 | Flow of participants or cases, using a diagram to indicate inclusion and exclusion | #33: Specify the criteria to include and exclude patients or examinations or pieces of information and document the numbers of cases that met each criterion. We strongly recommend including a flowchart/diagram in your results to show initial patient population and those excluded for any reason. | 0. Not documented  1. Documented |
|  | 34 | Demographic and clinical characteristics of cases in each partition | #34: Demographic and clinical characteristics of cases in each partition should be specified. State the performance metrics on all data partitions. | 0. Documented  D. Documented aggregate statistics  DP. Documented statistics for each data partition |
| **Model performance** | 35a | Test performance | #35: Report the final model’s performance on the test partition. 0 | 0. Not documented  V. Performance on validation dataset  T. Performance on testing dataset |
|  | 35b | Benchmark of performance |  | 0. Not documented  1. Documented |
|  | 36 | Estimates of diagnostic accuracy and their precision (such as 95% confidence intervals) | #36: For classification tasks, include estimates of diagnostic accuracy and their precision, such as 95% confidence intervals. | 0. Diagnostic performance reported without measure of precision  1. Diagnostic performance reported with confidence interval or standard error |
|  | 37 | Failure analysis of incorrectly classified cases | #37: Provide information to help understand incorrect results. If the task entails classification into two or more categories, provide a confusion matrix that shows tallies for predicted versus actual categories. Consider presenting examples of incorrectly classified cases to help readers better understand the strengths and limitations of the algorithm. | 0. Not discussed  1. Discussed misclassified cases or model errors |
| **DISCUSSION** | | | | |
| **Study limitations** | 38 | Study limitations, including potential bias, statistical uncertainty, and generalizability | #38: Summarize the results succinctly and place them into context; explain how the current work advances our knowledge and the state of the art. Identify the study’s limitations, including those involving the study’s methods, materials, biases, statistical uncertainty, unexpected results, and generalizability. | 0. Not discussed  1. Discussed |
| **Implications for practice** | 39 | Implications for practice, including the intended use and/or clinical role | #39: Describe the implications for practice, including the intended use and possible clinical role of the AI model. Describe the key impact the work may have on the field. Envision the next steps that one might take to build upon the results. Discuss any issues that would impede successful translation of the model into practice. | 0. Not discussed  1. Discussed |
| **OTHER INFORMATION** | | | | |
| **Registration** | 40 | Registration number and name of registry | #40: Comply with the clinical trial registration statement from the International Committee of Medical Journal Editors (ICMJE). | 0. Not documented  1. Documented |
| **Study protocol** | 41 | Where the full study protocol can be accessed | #41: State where readers can access the full study protocol if it exceeds the journal’s word limit. | 0. Not documented  1. Provided access to the full study protocol |
| **Funding** | 42 | Sources of funding and other support; role of funders | #42: Specify the sources of funding and other support and the exact role of the funders in performing the study. Indicate whether the authors had independence in each phase of the study. | 0. Not documented  F. Funding source documented  FR. Funding source and role documented  NF. Stated no funding received |

Note: CLAIM = Checklist for Artificial Intelligence in Medical Imaging. Extracted from Mongan J, Moy L, Kahn CE Jr. Checklist for Artificial Intelligence in Medical Imaging (CLAIM): A Guide for Authors and Reviewers. Radiol Artif Intell. 2020 Mar 25;2(2):e200029

**Supplementary Table S5 QUADAS-2 tool for risk of bias and concern on application**

| **Domain and Description** | **Modified signaling question** | **Risk of bias** | **Applicability concern** |
| --- | --- | --- | --- |
| **Patient selection** - describe methods of patient selection: Describe included patients (prior testing, presentation, intended use of index test and setting) | Signaling question 1: was a consecutive or random sample of patients enrolled? | Could the selection of patients have introduced bias? | Are there concerns that the included patients do not match the review question? |
|  | Signaling question 2: was a case–control design avoided? |  |  |
|  | Signaling question 3: did the study avoid inappropriate exclusions? |  |  |
| **Index test** - describe the index test and how it was conducted and interpreted | Signaling question 1: were the imaging acquisition protocol, image processing approach described in detail? | Could the conduct or interpretation of the index test have introduced bias? | Are there concerns that the index test, its conduct, or interpretation differ from the review question? |
|  | Signaling question 2: were the segmentation method(s), and feature extraction software described in detail? |  |  |
|  | Signaling question 3: was the validation independent (i. e. external)? |  |  |
| **Reference standard** - describe the reference standard and how it was conducted and interpreted | Signaling question 1: is the reference standard likely to correctly classify the target condition? | Could the reference standard, its conduct, or its interpretation have introduced bias? | Are there concerns that the target condition as defined by the reference standard does not match the review question? |
| **Flow and timing** - describe any patients who did not receive the index test(s) and/or reference standard or who were excluded from the 2x2 table (refer to flow diagram): Describe the time interval and any interventions between index test(s) and reference standard | Signaling question 1: was there an appropriate interval between imaging and reference standard? | Could the patient flow have introduced bias? | Not applicable |

Note: QUADAS-2 = modified Quality Assessment of Diagnostic Accuracy Studies. Extracted from Whiting PF, Rutjes AW, Westwood Me, et al; QUADAS-2 Group. QUADAS-2: a revised tool for the quality assessment of diagnostic accuracy studies. Ann Intern Med. 2011;155(8):529-536.

**Supplementary Table S6 Types of Prediction Model Studies Covered by The TRIPOD Statement**

| **Model type** | **Description** |
| --- | --- |
| Type 1a | Development of a prediction model where predictive performance is then directly evaluated using exactly the same data (apparent performance). |
| Type 1b | Development of a prediction model using the entire data set, but then using resampling (e.g., bootstrapping or cross-validation) techniques to evaluate the performance and optimism of the developed model. Resampling techniques, generally referred to as “internal validation”, are recommended as a prerequisite for prediction model development, particularly if data are limited. |
| Type 2a | The data are randomly split into 2 groups: one to develop the prediction model and one to evaluate its predictive performance. This design is generally not recommended or better than type 1b, particularly in case of limited data, because it leads to lack of power during model development and validation. |
| Type 2b | The data are nonrandomly split (e.g., by location or time) into 2 groups: one to develop the prediction model and one to evaluate its predictive performance. Type 2b is a stronger design for evaluating model performance than type 2a because it allows for nonrandom variation between the 2 data sets. |
| Type 3 | Development of a prediction model using 1 data set and an evaluation of its performance on separate data (e.g., from a different study). |
| Type 4 | The evaluation of the predictive performance of an existing (published) prediction model on separate data. |

Note: TRIPOD = Transparent Reporting of a multivariable prediction model for Individual Prognosis Or Diagnosis. Extracted from Collins GS, Reitsma JB, Altman DG, Moons KG. Transparent reporting of a multivariable prediction model for individual prognosis or diagnosis (TRIPOD): the TRIPOD statement. Ann Intern Med. 2015;162(1):55-63.

**Supplementary Table S7 Trials Classifications for Image Mining Tools Development Process**

| **Trial phase** | **Sample size** | **Type of study** | **Validation approach** | **Development stage** |
| --- | --- | --- | --- | --- |
| Discovery science | Algorithm development, phantom, simulated data | Experimental | Without validation | Preclinical |
| Phase 0 | < 100 patients | Retrospective | Internal validation | Pre-marketing |
| Phase I | < 100 patients | Retrospective | Validation | Pre-marketing |
| Phase II | > 100 patients | Retrospective | Validation | Pre-marketing |
| Phase III | > 100 patients | Prospective | Validation | Pre-marketing |
| Phase IV | n/a | Real-world | Monitoring | Post-marketing |

Note: The validation process may be internal (e.g., cross-validation and bootstrapping) or external (using data not used for training). The external validation may be performed using three different strategies: (i) temporal (i.e., data obtained in newly recruited patients), (ii) geographic (i.e., data collected in a different institution), and (iii) split-sample (i.e., data split from the entire dataset and kept untouched for the test). Extracted form Sollini M, Antunovic L, Chiti A, Kirienko M. Towards clinical application of image mining: a systematic review on artificial intelligence and radiomics. Eur J Nucl Med Mol Imaging. 2019 Dec;46(13):2656-2672.

**Supplementary Table S8 Study characteristics of included studies**

| **Study** | **Year** | **Country** | **Journal** | **Impact Factor** | **Journal Type** | **First Authorship** | **Study Design** | **Imaging Modality** | **Biomarker** | **Clinical question** |
| --- | --- | --- | --- | --- | --- | --- | --- | --- | --- | --- |
| Nie2021 [19] | 2021 | China | Radiologic Practice | n/a | Imaging | Radiologist | Retrospective | CT | Diagnosis | GCTB vs. chordoma |
| Shi2017 [20] | 2017 | China | Chinese Journal of Medical Imaging Technology | n/a | Imaging | Radiologist | Retrospective | CT | Diagnosis | GCTB vs. aneurysmal bone cyst |
| Wang2021 [21] | 2021 | China | J Bone Oncol | 4.491 | Non-imaging | Radiologist | Retrospective | CT | Prognosis | Early recurrence in spinal GCTB |
| Wang2022 [22] | 2022 | China | Front Oncol | 5.738 | Non-imaging | Radiologist | Retrospective | MRI | Prediction | Expression of p53 and VEGF in GCTB |
| Wu2021 [23] | 2021 | China | Journal of Shenyang Medical College | n/a | Non-imaging | Radiologist | Retrospective | MRI | Diagnosis | GCTB vs. aneurysmal bone cyst |
| Yin2019A [24] | 2019 | China | Eur Radiol | 7.034 | Imaging | Radiologist | Retrospective | CT | Diagnosis | GCTB vs. chordoma in sacrum |
| Yin2019B [25] | 2019 | China | J Magn Reson Imaging | 5.119 | Imaging | Radiologist | Retrospective | MRI | Diagnosis | GCTB vs. chordoma vs. metastatic tumor in sacrum |
| Yin2019C [26] | 2019 | China | Br J Radiol | 3.629 | Imaging | Radiologist | Retrospective | CT + MRI | Diagnosis | GCTB vs. chordoma in sacrum |
| Yin2021 [27] | 2021 | China | Front Oncol | 5.738 | Non-imaging | Radiologist | Retrospective | CT | Diagnosis | GCTB vs. neurogenic tumor in pelvic and sacral tumor |

Note: GCTB = giant cell tumor of bone, ABC = aneurysmal bone cyst

**Supplementary Table S9 PICOT of included studies**

| **Study** | **Sample Size** | **Institution** | **Inclusion Period** | **Patient Condition** | **Gender (F/M)** | **Age** | **Comparing Test** | **Reference Standard** | **Outcome** | **Timing** |
| --- | --- | --- | --- | --- | --- | --- | --- | --- | --- | --- |
| Nie2021 [19] | 92 (33 GCTB + 59 chordoma) | Local data; two centers | 2010 Jul to 2020 Jul | pathologically proven GCTB and chordoma | 22/11; 25/34 | Median 33, 19-62; median 59, 25-83 y/o | None | Histology | GCTB vs. chordoma | 1 time point: pre-treatment |
| Shi2017 [20] | 43 (34 GCTB + 9 ABC) | Local data; two centers | 2015 Mar to 2016 Apr | pathologically proven GCTB and ABC | 18/16; 4/5 | 31.2 ± 11.1; 18.2 ± 7.2 y/o | None | Histology | GCTB vs. aneurysmal bone cyst | 1 time point: pre-treatment |
| Wang2021 [21] | 62 GCTB | Local data; one center | 2008 Mar to 2018 Feb | pathologically proven GCTB | Recurrence 23/22; non-recurrence 10/7 | Recurrence 31.9 ± 14.0; non-recurrence 32.7 ± 10.8 y/o | None | Follow up | Early recurrence in spinal GCTB | 1 time point: pre-treatment |
| Wang2022 [22] | 80 GCTB | Local data; one center | 2009 Apr to 2019 Jan | pathologically proven GCTB | 45/35 | High VEGF 33.3 ±13.3, low VEGF 32.2 ± 10.7; wild p53 32.6 ± 12.0, mutant p53 34.3 ± 14.0 y/o | SINS score, Enneking stage | Immunohistochemical staining | Expression of p53 and VEGF in GCTB | 1 time point: pre-treatment |
| Wu2021 [23] | 29 (16 GCTB + 13 ABC) | Local data; one center | 2010 Jan to 2019 May | pathologically proven GCTB and ABC | 6/10; 4/9 | 40.6 ± 19.6; 25.6 ± 16.6 y/o | Radiologists’ assessment | Histology | GCTB vs. aneurysmal bone cyst | 1 time point: pre-treatment |
| Yin2019A [24] | 95 (42 GCTB + 53 chordoma) | Local data; one center | 2006 Jan to 2017 Oct | pathologically proven GCTB and chordoma | 41/54 | 47.9 ± 17.1 | None | Histology | GCTB vs. chordoma in sacrum | 1 time point: pre-treatment |
| Yin2019B [25] | 120 (30 GCTB + 54 + chordoma + 36 metastatic tumors | Local data; one center | 2006 Jan 2017 Oct | pathologically proven GCTB, chordoma, and metastatic tumor | 57/63 | 50.9 ± 15.8 | None | Histology | GCTB vs. chordoma vs. metastatic tumor in sacrum | 1 time point: pre-treatment |
| Yin2019C [26] | 137 (54 GCTB + 83 chordoma) | Local data; one center | 2006 Jan to 2018 Jun | pathologically proven GCTB and chordoma | 33/21; 27/56 | 35.2 ± 11.3; 55.8 ± 14.2 | None | Histology | GCTB vs. chordoma in sacrum | 1 time point: pre-treatment |
| Yin2021 [27] | 215 (120 GCTB + 95 neurogenic tumors) | Local data; one center | 2006 Apr to 2019 Dec | pathologically proven GCTB and neurogenic tumor | 54/66; 44/51 | Median 44, 32-53.8; median 33, 25-43 | None | Histology | GCTB vs. neurogenic tumor in pelvic and sacral tumor | 1 time point: pre-treatment |

Note: GCTB = giant cell tumor of bone, ABC = aneurysmal bone cyst, SINS = Spinal Instability Neoplastic Score

**Supplementary Table S10 Radiomics methodological issue of included studies**

| **Study** | **Imaging** | **ROI segmentation** | **Radiomics feature extraction** | **Non-radiomics features** | **Feature reduction and selection** | **Selected features** | **Classifier/ Evaluation** | **Outcome** | **Validation** |
| --- | --- | --- | --- | --- | --- | --- | --- | --- | --- |
| Nie2021 [19] | CT | CM; 2 URs, manual; ITK-SNAP v3.8; ICC | Radiomics cloud platform (Huiying Medical Technology Co., Ltd) | Clinical parameters | ICC for reproducibility; LASSO, 10-fold cross-validation | 4 FO, 1 GLCM, 2 GLSZM | LASSO; AUC, Hosmer-Lemeshow test, DCA | GCTB vs. chordoma | External validation (3) (64/28) |
| Shi2017 [20] | CT | C; not documented; Image J; None | Image J | None | None | 2 FO | None; AUC | GCTB vs. aneurysmal bone cyst | The same dataset (1a) |
| Wang2021 [21] | CT | C; 2 SRs, manual; not documented; None | Pyradiomics, Python v3.6 | None | SVM with Gaussian kernel, 10-fold cross-validation | 4 FO, 2 GLCM, 1 GLSZM, 1 CLRLM, 2 GLDM | SVM with Gaussian kernel, 10-fold cross-validation; AUC | Early recurrence in spinal GCTB | 10-fold cross-validation (1b) |
| Wang2022 [22] | MRI | C; 2 SRs, manual; Image J; None | Pyradiomics | Clinical parameters | SVM with Gaussian kernel, RF, 10-fold cross-validation | 1 Shape, 1 FO, 1 GLCM | LR; AUC | Expression of p53 and VEGF in GCTB | 10-fold cross-validation (1b) |
| Wu2021 [23] | MRI | CM; 2 URs, manual; ITK-SNAP; ICC | Artificial Intelligence Kit (GE Healthcare) | Radiologists’ assessment | Spearman correlation | 1 GLCM, 2 GLRLM | LR; AUC, Hosmer-Lemeshow test | GCTB vs. aneurysmal bone cyst | The same dataset (1a) |
| Yin2019A [24] | CT | CM; 2 SRs, manual; ITK-SNAP; ICC | Artificial Intelligence Kit, v1.0.3 (GE Healthcare) | None | Relief, LASSO, RF | not documented | SVM, GLM, LR, 10-fold cross-validation; AUC, accuracy | GCTB vs. chordoma in sacrum | Internal validation (2a) (66/29) |
| Yin2019B [25] | MRI | CM; 2 URs, manual; ITK-SNAP; ICC | Artificial Intelligence Kit, v1.0.3 (GE Healthcare) | None | ANOVA, LASSO, Pearson correlation, RF, 10-fold cross-validation | 1 Shape, 3 FO, 3 GLCM, 3 GLRLM for T2FS model; 1 Shape, 1 FO, 5 GLCM, 3 GLRLM for T1CE; 1 Shape, 3 FO, 3 GLCM, 3 GLRLM for T2FS + T1CE; | RF, 10-fold cross-validation; AUC, accuracy | GCTB vs. chordoma vs. metastatic tumor in sacrum | Internal validation (2a) (83/37) |
| Yin2019C [26] | CT + MRI | CM; 2 SRs, manual; ITK-SNAP; ICC | Artificial Intelligence Kit, v1.0.3 (GE Healthcare) | Clinical parameters | LASSO, GLM, Spearman correlation | 4 FO, 5 GLCM, 1 CLRLM for MRI; 1 FO, 7 GLCM for CT | LASSO, 10-fold cross-validation; AUC, accuracy | GCTB vs. chordoma in sacrum | Internal validation (2a) (96/41) |
| Yin2021 [27] | CT | CM; 2 SRs, semi-automatic; MITK; ICC | Artificial Intelligence Kit, v3.3.0 (GE Healthcare) | Clinical parameters | GLBT, Spearman correlation | not documented | RF, 5- fold cross-validation; AUC, accuracy | GCTB vs. neurogenic tumor in pelvic and sacral tumor | Internal validation (2a) (7:3 = 150.5: 64.5) |

Note: C = Image cropping documented, CM = Reproducible image cropping method documented; UR = Radiologist with unspecified expertise, SR = Radiologist with relevant subspecialist expertise, LASSO = least absolute shrinkage and selection operator, SVM = support vector machine, RF = random forest, GLM = generalised linear model, GBDT = gradient boosting decision tree, LR = logistic regression, ANOVA = analysis of variance, ICC = interclass coefficient.

**Supplementary Table S11 Model presentation and performance metrics of included studies**

| **Study** | **Full prediction model** | **Nomogram, calculator, etc.** | **Biological correlations** | **Discrimination statistics** | **Performance** | **Calibration statistics** | **Cost-effectiveness analysis** | **Potential clinical utility** | **Open science** |
| --- | --- | --- | --- | --- | --- | --- | --- | --- | --- |
| Nie2021 [19] | Yes | Nomogram, formula | Tumor heterogeneity | AUC with 95%CI | 0.908 (0.800-0.980) for training; 0.860 (0.700-1.000) for validation | Hosmer-Lemeshow test | None | DCA | None |
| Shi2017 [20] | No | None | Tumor component (cyst – ABC, osteoclast cell- GCTB) | AUC with P value | 0.96 (P<0.01) for average CT value; 0.95 (P <0.01) for peak CT value | None | None | No | None |
| Wang2021 [21] | No | None | Tumor heterogeneity | AUC with P value | 0.78 for final model; 0.62-0.78 for individual feature | None | None | No | Open-source radiomics extraction software |
| Wang2022 [22] | No | None | Tumor heterogeneity; genetic | AUC with 95%CI | 0.781 (0.676-0.886) for VEGF; 0.737 (0.562-0.913) for p53 | None | None | No | Open-source radiomics extraction software |
| Wu2021 [23] | Yes | Coefficient provided | None | AUC with 95%CI | 0.890 for final model; 0.110-0.890 for individual feature | Hosmer-Lemeshow test | None | No | None |
| Yin2019A [24] | No | None | None | AUC with 95%CI | 0.834-1.000 for training; 0.639-0.984 for validation | None | None | No | None |
| Yin2019B [25] | No | None | Rough bound, Elongated shape of tumor | AUC only | 0.773 for training; 0.643 for validation | None | None | No | None |
| Yin2019C [26] | Yes | Nomogram, formula | None | AUC only | 0.990 for CT training; 0.948 for CT validation; 0.996 for MRI training; 0.870 for MRI validation | Calibration curve | None | DCA | None |
| Yin2021 [27] | No | None | None | AUC only | 0.899 for training; 0.928 for validation | None | None | No | None |

Note: AUC = aera under curve, DCA = decision curve analysis.

**Supplementary Table S12 RQS rating per study**

| **Study** | **Nie2021 [19]** | **Shi2017 [20]** | **Wang2021 [21]** | **Wang2022 [22]** | **Wu2021 [23]** | **Yin2019A [24]** | **Yin2019B [25]** | **Yin2019C [26]** | **Yin2021 [27]** |
| --- | --- | --- | --- | --- | --- | --- | --- | --- | --- |
| **Total 16 items (ideal score 36)** | 16 | -2 | 6 | 9 | 9 | 11 | 11 | 13 | 11 |
| **Domain 1: protocol quality and stability in image and segmentation (0 to 5 points)** | 2 | 1 | 2 | 2 | 2 | 2 | 2 | 2 | 2 |
| **Protocol quality (2 points)** | 1 | 1 | 1 | 1 | 1 | 1 | 1 | 1 | 1 |
| **Multiple segmentations (1 point)** | 1 | 0 | 1 | 1 | 1 | 1 | 1 | 1 | 1 |
| **Test-retest (1 point)** | 0 | 0 | 0 | 0 | 0 | 0 | 0 | 0 | 0 |
| **Phantom study (1 point)** | 0 | 0 | 0 | 0 | 0 | 0 | 0 | 0 | 0 |
| **Domain 2: feature selection and validation (-8 to 8 points)** | 6 | -8 | -2 | -2 | -2 | 5 | 5 | 5 | 5 |
| **Feature reduction or adjustment of multiple testing (-3 or 3 points)** | 3 | -3 | 3 | 3 | 3 | 3 | 3 | 3 | 3 |
| **Validation (-5, 2, 3, 4, or 5 points)** | 3 | -5 | -5 | -5 | -5 | 2 | 2 | 2 | 2 |
| **Domain 3: biologic/clinical validation and utility (0 to 6 points)** | 4 | 3 | 3 | 6 | 5 | 2 | 3 | 3 | 3 |
| **Non-radiomics features (1 point)** | 1 | 0 | 0 | 1 | 1 | 0 | 0 | 1 | 1 |
| **Biologic correlations (1 point)** | 1 | 1 | 1 | 1 | 0 | 0 | 1 | 0 | 0 |
| **Comparison to “gold standard” (2 points)** | 0 | 0 | 0 | 2 | 2 | 0 | 0 | 0 | 0 |
| **Potential clinical utility (2 points)** | 2 | 2 | 2 | 2 | 2 | 2 | 2 | 2 | 2 |
| **Domain 4: model performance index (0 to 5 points)** | 4 | 2 | 2 | 2 | 4 | 2 | 1 | 3 | 1 |
| **Cut-off analysis (1 point)** | 0 | 0 | 0 | 0 | 0 | 0 | 0 | 0 | 0 |
| **Discrimination statistics (2 points)** | 2 | 2 | 2 | 2 | 2 | 2 | 1 | 1 | 1 |
| **Calibration statistics (2 points)** | 2 | 0 | 0 | 0 | 2 | 0 | 0 | 2 | 0 |
| **Domain 5: high level of evidence (0 to 8 points)** | 0 | 0 | 0 | 0 | 0 | 0 | 0 | 0 | 0 |
| **Prospective study (7 points)** | 0 | 0 | 0 | 0 | 0 | 0 | 0 | 0 | 0 |
| **Cost-effectiveness analysis (1 point)** | 0 | 0 | 0 | 0 | 0 | 0 | 0 | 0 | 0 |
| **Domain 6: Open science and data (0 to 4 points)** | 0 | 0 | 1 | 1 | 0 | 0 | 0 | 0 | 0 |

Note: RQS = Radiomics Quality Score. Extracted from Lambin P, Leijenaar RTH, Deist TM, et al. Radiomics: the bridge between medical imaging and personalized medicine. Nat Rev Clin Oncol. 2017;14(12):749-762.

**Supplementary Table S13 TRIPOD adherence per study**

| **Study** | **Nie2021 [19]** | **Shi2017 [20]** | **Wang2021 [21]** | **Wang2022 [22]** | **Wu2021 [23]** | **Yin2019A [24]** | **Yin2019B [25]** | **Yin2019C [26]** | **Yin2021 [27]** |
| --- | --- | --- | --- | --- | --- | --- | --- | --- | --- |
| **Overall** | 19 | 10 | 17 | 17 | 16 | 15 | 15 | 16 | 17 |
| **Title and Abstract** | 1 | 0 | 1 | 0 | 0 | 0 | 1 | 1 | 0 |
| **1** | 0 | 0 | 0 | 0 | 0 | 0 | 0 | 0 | 0 |
| **2** | 1 | 0 | 1 | 0 | 0 | 0 | 1 | 1 | 0 |
| **Introduction** | 2 | 1 | 2 | 1 | 1 | 1 | 2 | 2 | 1 |
| **3a** | 1 | 1 | 1 | 1 | 1 | 1 | 1 | 1 | 1 |
| **3b** | 1 | 0 | 1 | 0 | 0 | 0 | 1 | 1 | 0 |
| **Methods** | 9 | 5 | 8 | 9 | 9 | 8 | 8 | 8 | 9 |
| **4a** | 1 | 1 | 1 | 1 | 1 | 1 | 1 | 1 | 1 |
| **4b** | 1 | 1 | 1 | 1 | 1 | 1 | 1 | 1 | 1 |
| **5a** | 1 | 1 | 1 | 1 | 1 | 1 | 1 | 1 | 1 |
| **5b** | 1 | 0 | 1 | 1 | 1 | 1 | 1 | 1 | 1 |
| **5c, if relevant (N = 1)** | n/a | n/a | 1 | n/a | n/a | n/a | n/a | n/a | n/a |
| **6a** | 1 | 1 | 1 | 1 | 1 | 1 | 1 | 1 | 1 |
| **6b** | 0 | 0 | 0 | 0 | 0 | 0 | 0 | 0 | 0 |
| **7a** | 1 | 0 | 1 | 1 | 1 | 1 | 1 | 1 | 1 |
| **7b** | 0 | 0 | 0 | 1 | 0 | 0 | 0 | 0 | 1 |
| **8** | 0 | 0 | 0 | 0 | 0 | 0 | 0 | 0 | 0 |
| **9** | 0 | 0 | 0 | 0 | 0 | 0 | 0 | 0 | 0 |
| **10a** | 1 | 1 | 1 | 1 | 1 | 1 | 1 | 1 | 1 |
| **10b** | 1 | 0 | 1 | 1 | 1 | 1 | 1 | 1 | 1 |
| **10d** | 1 | 0 | 0 | 0 | 1 | 0 | 0 | 0 | 0 |
| **11, if done (N = 1)** | n/a | n/a | n/a | 1 | n/a | n/a | n/a | n/a | n/a |
| **Results** | 4 | 2 | 3 | 4 | 3 | 2 | 1 | 2 | 4 |
| **13a** | 0 | 0 | 1 | 1 | 0 | 0 | 0 | 0 | 1 |
| **13b** | 0 | 0 | 0 | 1 | 0 | 0 | 0 | 1 | 1 |
| **14a** | 1 | 1 | 1 | 1 | 1 | 1 | 1 | 1 | 0 |
| **14b, if done (N = 1)** | n/a | n/a | n/a | n/a | 1 | n/a | n/a | n/a | n/a |
| **15a** | 1 | 0 | 0 | 0 | 1 | 0 | 0 | 0 | 1 |
| **15b** | 1 | 0 | 0 | 0 | 0 | 0 | 0 | 0 | 1 |
| **16** | 1 | 1 | 1 | 1 | 1 | 1 | 0 | 0 | 0 |
| **Discussion** | 3 | 2 | 3 | 3 | 3 | 3 | 3 | 3 | 3 |
| **18** | 1 | 0 | 1 | 1 | 1 | 1 | 1 | 1 | 1 |
| **19b** | 1 | 1 | 1 | 1 | 1 | 1 | 1 | 1 | 1 |
| **20** | 1 | 1 | 1 | 1 | 1 | 1 | 1 | 1 | 1 |
| **Other information** | 0 | 0 | 0 | 0 | 0 | 1 | 0 | 0 | 0 |
| **21** | 0 | 0 | 0 | 0 | 0 | 0 | 0 | 0 | 0 |
| **22** | 0 | 0 | 0 | 0 | 0 | 1 | 0 | 0 | 0 |
| **Validation (N = 5)** | 3 | n/a | n/a | n/a | n/a | 2 | 2 | 0 | 1 |
| **Model type** | 3 | 1a | 1b | 1b | 1a | 2a | 2a | 2a | 2a |
| **10c** | 1 | n/a | n/a | n/a | n/a | 0 | 0 | 0 | 1 |
| **10e, if done (N = 0)** | n/a | n/a | n/a | n/a | n/a | n/a | n/a | n/a | n/a |
| **12** | 1 | n/a | n/a | n/a | n/a | 1 | 1 | 0 | 0 |
| **13c** | 1 | n/a | n/a | n/a | n/a | 1 | 1 | 0 | 0 |
| **17, if done (N = 0)** | n/a | n/a | n/a | n/a | n/a | n/a | n/a | n/a | n/a |
| **19a** | 0 | n/a | n/a | n/a | n/a | 0 | 0 | 0 | 0 |

Note: TRIPOD = Transparent Reporting of a multivariable prediction model for Individual Prognosis Or Diagnosis. Extracted from Collins GS, Reitsma JB, Altman DG, Moons KG. Transparent reporting of a multivariable prediction model for individual prognosis or diagnosis (TRIPOD): the TRIPOD statement. Ann Intern Med. 2015;162(1):55-63.

**Supplementary Table S14 CLAIM adherence per study**

| **Study** | **Nie2021 [19]** | **Shi2017 [20]** | **Wang2021 [21]** | **Wang2022 [22]** | **Wu2021 [23]** | **Yin2019A [24]** | **Yin2019B [25]** | **Yin2019C [26]** | **Yin2021 [27]** |
| --- | --- | --- | --- | --- | --- | --- | --- | --- | --- |
| **Overall** | 33 | 18 | 30 | 31 | 26 | 34 | 29 | 31 | 30 |
| **Title and Abstract** | 2 | 1 | 2 | 2 | 2 | 2 | 1 | 2 | 1 |
| **1** | 1 | 1 | 1 | 1 | 1 | 1 | 1 | 1 | 1 |
| **2** | 1 | 0 | 1 | 1 | 1 | 1 | 0 | 1 | 0 |
| **Introduction** | 2 | 2 | 2 | 2 | 2 | 2 | 2 | 2 | 2 |
| **3** | 1 | 1 | 1 | 1 | 1 | 1 | 1 | 1 | 1 |
| **4a** | 1 | 1 | 1 | 1 | 1 | 1 | 1 | 1 | 1 |
| **4b** | 0 | 0 | 0 | 0 | 0 | 0 | 0 | 0 | 0 |
| **Methods** | 25 | 13 | 22 | 21 | 18 | 26 | 23 | 23 | 22 |
| **5** | 1 | 1 | 1 | 1 | 1 | 1 | 1 | 1 | 1 |
| **6** | 1 | 1 | 1 | 1 | 1 | 1 | 1 | 1 | 1 |
| **7a** | 1 | 1 | 1 | 1 | 1 | 1 | 1 | 1 | 1 |
| **7b** | 1 | 1 | 1 | 1 | 1 | 1 | 1 | 1 | 1 |
| **7c** | 1 | 1 | 1 | 1 | 1 | 1 | 1 | 1 | 1 |
| **7d** | 1 | 1 | 1 | 1 | 1 | 1 | 1 | 1 | 1 |
| **7e** | 1 | 0 | 1 | 1 | 0 | 1 | 1 | 1 | 1 |
| **7f** | 0 | 0 | 1 | 1 | 0 | 1 | 1 | 1 | 0 |
| **8** | 1 | 0 | 1 | 1 | 1 | 1 | 1 | 1 | 1 |
| **9** | 0 | 0 | 0 | 0 | 0 | 0 | 0 | 0 | 1 |
| **10** | 1 | 0 | 1 | 1 | 1 | 1 | 1 | 1 | 1 |
| **11** | 1 | 1 | 1 | 1 | 1 | 1 | 1 | 1 | 1 |
| **12** | 0 | 0 | 0 | 0 | 0 | 0 | 0 | 0 | 0 |
| **13** | 0 | 0 | 0 | 0 | 0 | 0 | 0 | 0 | 0 |
| **14** | 1 | 1 | 1 | 1 | 1 | 1 | 1 | 1 | 1 |
| **15a, if alternatives exist (N = 0)** | n/a | n/a | n/a | n/a | n/a | n/a | n/a | n/a | n/a |
| **15b** | 1 | 1 | 1 | 1 | 1 | 1 | 1 | 1 | 1 |
| **16** | 0 | 0 | 1 | 1 | 0 | 1 | 0 | 1 | 1 |
| **17** | 1 | 0 | 0 | 0 | 0 | 0 | 0 | 0 | 0 |
| **18** | 1 | 0 | 0 | 0 | 1 | 1 | 1 | 1 | 1 |
| **19a** | 1 | 1 | 1 | 1 | 1 | 1 | 1 | 1 | 1 |
| **19b** | 0 | 0 | 0 | 0 | 0 | 0 | 0 | 0 | 0 |
| **19c** | 1 | 0 | 0 | 0 | 0 | 1 | 1 | 0 | 0 |
| **20** | 1 | 0 | 0 | 0 | 0 | 1 | 1 | 0 | 0 |
| **21** | 1 | 1 | 1 | 1 | 1 | 1 | 1 | 1 | 1 |
| **22a** | 1 | 0 | 1 | 1 | 1 | 1 | 1 | 1 | 1 |
| **22b** | 0 | 0 | 0 | 0 | 0 | 0 | 0 | 0 | 0 |
| **23** | 0 | 0 | 1 | 0 | 0 | 1 | 1 | 1 | 1 |
| **24** | 0 | 0 | 0 | 0 | 0 | 0 | 0 | 0 | 0 |
| **25** | 1 | 0 | 1 | 1 | 1 | 1 | 1 | 1 | 1 |
| **26** | 1 | 0 | 1 | 1 | 0 | 1 | 1 | 1 | 1 |
| **27, if applicable (N = 5)** | 1 | n/a | n/a | 1 | 1 | n/a | n/a | 1 | 1 |
| **28** | 1 | 1 | 1 | 1 | 1 | 1 | 1 | 1 | 1 |
| **29** | 1 | 1 | 1 | 1 | 1 | 1 | 0 | 0 | 0 |
| **30** | 0 | 0 | 0 | 0 | 0 | 1 | 0 | 0 | 0 |
| **31** | 1 | 0 | 0 | 0 | 0 | 0 | 0 | 1 | 0 |
| **32** | 1 | 0 | 0 | 0 | 0 | 0 | 0 | 0 | 0 |
| **Results** | 2 | 1 | 2 | 4 | 2 | 2 | 1 | 2 | 3 |
| **33** | 0 | 0 | 1 | 1 | 0 | 0 | 0 | 0 | 1 |
| **34** | 0 | 0 | 0 | 1 | 0 | 0 | 0 | 1 | 1 |
| **35a** | 1 | 0 | 0 | 0 | 0 | 1 | 1 | 1 | 1 |
| **35b** | 0 | 0 | 0 | 1 | 1 | 0 | 0 | 0 | 0 |
| **36** | 1 | 1 | 1 | 1 | 1 | 1 | 0 | 0 | 0 |
| **37** | 0 | 0 | 0 | 0 | 0 | 0 | 0 | 0 | 0 |
| **Discussion** | 2 | 1 | 2 | 2 | 2 | 2 | 2 | 2 | 2 |
| **38** | 1 | 0 | 1 | 1 | 1 | 1 | 1 | 1 | 1 |
| **39** | 1 | 1 | 1 | 1 | 1 | 1 | 1 | 1 | 1 |
| **Other information** | 0 | 0 | 0 | 0 | 0 | 1 | 0 | 0 | 0 |
| **40** | 0 | 0 | 0 | 0 | 0 | 0 | 0 | 0 | 0 |
| **41** | 0 | 0 | 0 | 0 | 0 | 0 | 0 | 0 | 0 |
| **42** | 0 | 0 | 0 | 0 | 0 | 1 | 0 | 0 | 0 |

Note: CLAIM = Checklist for Artificial Intelligence in Medical Imaging. Extracted from Mongan J, Moy L, Kahn CE Jr. Checklist for Artificial Intelligence in Medical Imaging (CLAIM): A Guide for Authors and Reviewers. Radiol Artif Intell. 2020 Mar 25;2(2):e200029

**Supplementary Table S15 QUADAS-2 assessment per study**

| **Study** | **Nie2021 [19]** | **Shi2017 [20]** | **Wang2021 [21]** | **Wang2022 [22]** | **Wu2021 [23]** | **Yin2019A [24]** | **Yin2019B [25]** | **Yin2019C [26]** | **Yin2021 [27]** |
| --- | --- | --- | --- | --- | --- | --- | --- | --- | --- |
| **Risk of bias** | | | | | | | | | |
| **Patient Selection** | L | L | L | L | L | L | L | L | L |
| **Index Test** | L | H | H | H | H | H | H | H | H |
| **Reference Standard** | L | L | L | L | L | L | L | L | L |
| **Flow and Timing** | L | U | U | U | L | U | U | U | U |
| **Application concern** | | | | | | | | | |
| **Patient Selection** | L | L | L | L | L | L | L | L | L |
| **Index Test** | L | H | H | H | H | H | H | H | H |
| **Reference Standard** | L | L | L | L | L | L | L | L | L |

Note: QUADAS-2 = modified Quality Assessment of Diagnostic Accuracy Studies. L = low risk, U = unclear, H = high risk. Extracted from Whiting PF, Rutjes AW, Westwood Me, et al; QUADAS-2 Group. QUADAS-2: a revised tool for the quality assessment of diagnostic accuracy studies. Ann Intern Med. 2011;155(8):529-536.
