# Supplementary material for: A systematic review of radiomics in giant cell tumor of bone (GCTB): the potential of analysis on individual radiomics feature for identifying genuine promising imaging biomarkers
Source: J Orthop Surg Res. 2023 Jun 7;18:414. doi: 10.1186/s13018-023-03863-w (PMC10249293; doi:10.1186/s13018-023-03863-w)
Supplement: Supplementary file 2 — Additional file 2. PRISMA Checklists. [file 13018_2023_3863_MOESM2_ESM.docx]

| **Section and Topic** | **Item #** | **Checklist item** | **Location where item is reported** |
| --- | --- | --- | --- |
| **TITLE** | | |  |
| Title | 1 | Identify the report as a systematic review. | Page 1 |
| **ABSTRACT** | | |  |
| Abstract | 2 | See the PRISMA 2020 for Abstracts checklist. | n/a |
| **INTRODUCTION** | | |  |
| Rationale | 3 | Describe the rationale for the review in the context of existing knowledge. | Page 2 |
| Objectives | 4 | Provide an explicit statement of the objective(s) or question(s) the review addresses. | Page 2 |
| **METHODS** | | |  |
| Eligibility criteria | 5 | Specify the inclusion and exclusion criteria for the review and how studies were grouped for the syntheses. | Page 2, Supplementary Note S1 |
| Information sources | 6 | Specify all databases, registers, websites, organisations, reference lists and other sources searched or consulted to identify studies. Specify the date when each source was last searched or consulted. | Page 2, Supplementary Note S2 |
| Search strategy | 7 | Present the full search strategies for all databases, registers and websites, including any filters and limits used. | Page 2, Supplementary Note S2 |
| Selection process | 8 | Specify the methods used to decide whether a study met the inclusion criteria of the review, including how many reviewers screened each record and each report retrieved, whether they worked independently, and if applicable, details of automation tools used in the process. | Page 2, Supplementary Note S2 |
| Data collection process | 9 | Specify the methods used to collect data from reports, including how many reviewers collected data from each report, whether they worked independently, any processes for obtaining or confirming data from study investigators, and if applicable, details of automation tools used in the process. | Page 3, Supplementary Note S3 |
| Data items | 10a | List and define all outcomes for which data were sought. Specify whether all results that were compatible with each outcome domain in each study were sought (e.g. for all measures, time points, analyses), and if not, the methods used to decide which results to collect. | Page 3, Supplementary Table S1 |
|  | 10b | List and define all other variables for which data were sought (e.g. participant and intervention characteristics, funding sources). Describe any assumptions made about any missing or unclear information. | Page 3, Supplementary Table S1 |
| Study risk of bias assessment | 11 | Specify the methods used to assess risk of bias in the included studies, including details of the tool(s) used, how many reviewers assessed each study and whether they worked independently, and if applicable, details of automation tools used in the process. | Page 3, Supplementary Note S3, Supplementary Table S2 -S5 |
| Effect measures | 12 | Specify for each outcome the effect measure(s) (e.g. risk ratio, mean difference) used in the synthesis or presentation of results. | Page 3, Supplementary Note S4 |
| Synthesis methods | 13a | Describe the processes used to decide which studies were eligible for each synthesis (e.g. tabulating the study intervention characteristics and comparing against the planned groups for each synthesis (item #5)). | Page 3, Supplementary Note S4. Supplementary Tables S6-S7 |
|  | 13b | Describe any methods required to prepare the data for presentation or synthesis, such as handling of missing summary statistics, or data conversions. | Page 3, Supplementary Note S4 |
|  | 13c | Describe any methods used to tabulate or visually display results of individual studies and syntheses. | Page 3, Supplementary Note S4 |
|  | 13d | Describe any methods used to synthesize results and provide a rationale for the choice(s). If meta-analysis was performed, describe the model(s), method(s) to identify the presence and extent of statistical heterogeneity, and software package(s) used. | Page 3, Supplementary Note S4 |
|  | 13e | Describe any methods used to explore possible causes of heterogeneity among study results (e.g. subgroup analysis, meta-regression). | Page 3, Supplementary Note S4 |
|  | 13f | Describe any sensitivity analyses conducted to assess robustness of the synthesized results. | Page 3, Supplementary Note S4 |
| Reporting bias assessment | 14 | Describe any methods used to assess risk of bias due to missing results in a synthesis (arising from reporting biases). | Page 3, Supplementary Note S4 |
| Certainty assessment | 15 | Describe any methods used to assess certainty (or confidence) in the body of evidence for an outcome. | Page 3, Supplementary Note S4 |
| **RESULTS** | | |  |
| Study selection | 16a | Describe the results of the search and selection process, from the number of records identified in the search to the number of studies included in the review, ideally using a flow diagram. | Page 4-5, Figure 1-2 |
|  | 16b | Cite studies that might appear to meet the inclusion criteria, but which were excluded, and explain why they were excluded. | Page 4-5, Table 1-2, Supplementary Table S8-11 |
| Study characteristics | 17 | Cite each included study and present its characteristics. | Reference list |
| Risk of bias in studies | 18 | Present assessments of risk of bias for each included study. | Page 4-5, Table 3-5, Figure 3, Supplementary Table S12-S15 |
| Results of individual studies | 19 | For all outcomes, present, for each study: (a) summary statistics for each group (where appropriate) and (b) an effect estimate and its precision (e.g. confidence/credible interval), ideally using structured tables or plots. | Supplementary Table S12-S15 |
| Results of syntheses | 20a | For each synthesis, briefly summarise the characteristics and risk of bias among contributing studies. | Page 4-5, Table 3-5, Figure 3, Supplementary Table S12-S15 |
|  | 20b | Present results of all statistical syntheses conducted. If meta-analysis was done, present for each the summary estimate and its precision (e.g. confidence/credible interval) and measures of statistical heterogeneity. If comparing groups, describe the direction of the effect. | Page 4-5, Table 3-5, Figure 3, Supplementary Table S12-S15 |
|  | 20c | Present results of all investigations of possible causes of heterogeneity among study results. | Page 4-5, Table 3-5, Figure 3, Supplementary Table S12-S15 |
|  | 20d | Present results of all sensitivity analyses conducted to assess the robustness of the synthesized results. | Page 4-5, Table 3-5, Figure 3, Supplementary Table S12-S15 |
| Reporting biases | 21 | Present assessments of risk of bias due to missing results (arising from reporting biases) for each synthesis assessed. | Page 4-5, Table 3-5, Figure 3, Supplementary Table S12-S15 |
| Certainty of evidence | 22 | Present assessments of certainty (or confidence) in the body of evidence for each outcome assessed. | n/a |
| **DISCUSSION** | | |  |
| Discussion | 23a | Provide a general interpretation of the results in the context of other evidence. | Page 4-5 |
|  | 23b | Discuss any limitations of the evidence included in the review. | Page 6 |
|  | 23c | Discuss any limitations of the review processes used. | Page 6 |
|  | 23d | Discuss implications of the results for practice, policy, and future research. | Page 4-6 |
| **OTHER INFORMATION** | | |  |
| Registration and protocol | 24a | Provide registration information for the review, including register name and registration number, or state that the review was not registered. | Page 3, Supplementary Note S1 |
|  | 24b | Indicate where the review protocol can be accessed, or state that a protocol was not prepared. | Page 3, Supplementary Note S1 |
|  | 24c | Describe and explain any amendments to information provided at registration or in the protocol. | Page 3, Supplementary Note S1 |
| Support | 25 | Describe sources of financial or non-financial support for the review, and the role of the funders or sponsors in the review. | Declaration |
| Competing interests | 26 | Declare any competing interests of review authors. | Declaration |
| Availability of data, code and other materials | 27 | Report which of the following are publicly available and where they can be found: template data collection forms; data extracted from included studies; data used for all analyses; analytic code; any other materials used in the review. | Supplementary Materials |

*From:*  Page MJ, McKenzie JE, Bossuyt PM, Boutron I, Hoffmann TC, Mulrow CD, et al. The PRISMA 2020 statement: an updated guideline for reporting systematic reviews. BMJ 2021;372:n71. doi: 10.1136/bmj.n71

For more information, visit: <http://www.prisma-statement.org/>

**PRISMA 2020 checklist for abstract**

| **TITLE** | **CHECKLIST ITEM** | **REPORTED ON PAGE #** |
| --- | --- | --- |
| 1. Title: | Identify the report as a systematic review, meta-analysis, or both. | Page 1 |
| BACKGROUND |  |  |
| 2. Objectives: | The research question including components such as participants, interventions, comparators, and outcomes. | Page 1 |
| METHODS |  |  |
| 3. Eligibility criteria: | Study and report characteristics used as criteria for inclusion. | Page 1 |
| 4. Information sources: | Key databases searched and search dates. | Page 1 |
| 5. Risk of bias: | Methods of assessing risk of bias. | Page 1 |
| RESULTS |  |  |
| 6. Included studies: | Number and type of included studies and participants and relevant characteristics of studies. | Page 1 |
| 7. Synthesis of results: | Results for main outcomes (benefits and harms), preferably indicating the number of studies and participants for each. If meta-analysis was done, include summary measures and confidence intervals. | Page 1 |
| 8. Description of the effect: | Direction of the effect (i.e. which group is favoured) and size of the effect in terms meaningful to clinicians and patients. | Page 1 |
| DISCUSSION |  |  |
| 9. Strengths and Limitations of evidence: | Brief summary of strengths and limitations of evidence (e.g. inconsistency, imprecision, indirectness, or risk of bias, other supporting or conflicting evidence) | n/a |
| 10. Interpretation: | General interpretation of the results and important implications | Page 1 |
| OTHER |  |  |
| 11. Funding: | Primary source of funding for the review. | Declaration |
| 12. Registration: | Registration number and registry name. | Page 3 |
