# Supplementary material for: A systematic review of radiomics in giant cell tumor of bone (GCTB): the potential of analysis on individual radiomics feature for identifying genuine promising imaging biomarkers
Source: J Orthop Surg Res. 2023 Jun 7;18:414. doi: 10.1186/s13018-023-03863-w (PMC10249293; doi:10.1186/s13018-023-03863-w)
Supplement: Supplementary file 3 — Additional file 3. PROSPERO Review Protocol. [file 13018_2023_3863_MOESM3_ESM.pdf]

National Institute for Health Research

UNIVERSITY of York
Centre for Reviews and Dissemination

Systematic review

A list of fields that can be edited in an update can be found here

1. * Review title.

Give the title of the review in English

Radiomics in giant cell tumor of bone (GCTB): a systematic review

2. Original language title.

For reviews in languages other than English, give the title in the original language. This will be displayed with the English language title.

3. * Anticipated or actual start date.

Give the date the systematic review started or is expected to start.

15/07/2022

4. * Anticipated completion date.

Give the date by which the review is expected to be completed.

31/12/2022

5. * Stage of review at time of this submission.

This field uses answers to initial screening questions. It cannot be edited until after registration.

Tick the boxes to show which review tasks have been started and which have been completed.

Update this field each time any amendments are made to a published record.

The review has not yet started: No

Review stage	Started	Completed
Preliminary searches	Yes	No
Piloting of the study selection process	Yes	No
Formal screening of search results against eligibility criteria	No	No
Data extraction	No	No
Risk of bias (quality) assessment	No	No
Data analysis	No	No

Provide any other relevant information about the stage of the review here.

6. * Named contact.

The named contact is the guarantor for the accuracy of the information in the register record. This may be any member of the review team.

Jingyu Zhong

Email salutation (e.g. "Dr Smith" or "Joanne") for correspondence:

Dr Zhong

7. * Named contact email.

Give the electronic email address of the named contact.

wal_zjy@163.com

8. Named contact address

Give the full institutional/organisational postal address for the named contact.

No. 1111, Xianxia Rd., Changning District, Shanghai 200336, China

9. Named contact phone number.

Give the telephone number for the named contact, including international dialling code.

15221992711

10. * Organisational affiliation of the review.

Full title of the organisational affiliations for this review and website address if available. This field may be completed as 'None' if the review is not affiliated to any organisation.

Department of Imaging, Tongren Hospital, Shanghai Jiao Tong University School of Medicine

Organisation web address:

11. * Review team members and their organisational affiliations.

Give the personal details and the organisational affiliations of each member of the review team. Affiliation refers to groups or organisations to which review team members belong. **NOTE: email and country now MUST be entered for each person, unless you are amending a published record.**

Dr Jingyu Zhong. Department of Imaging, Tongren Hospital, Shanghai Jiao Tong University School of Medicine
Dr Yue Xing. Department of Imaging, Tongren Hospital, Shanghai Jiao Tong University School of Medicine
Professor Weiwu Yao. Department of Imaging, Tongren Hospital, Shanghai Jiao Tong University School of Medicine

12. * Funding sources/sponsors.

Details of the individuals, organizations, groups, companies or other legal entities who have funded or sponsored the review.

This study has received funding by Yangfan Project of Science and Technology Commission of Shanghai Municipality (22YF1442400); Shanghai Science and Technology Commission Science and Technology Innovation Action Clinical Innovation Field (18411953000); Medicine and Engineering Combination Project of Shanghai Jiao Tong University (YG2019ZDB09); and Research Fund of Tongren Hospital, Shanghai Jiao Tong University School of Medicine (TRKYRC-XX202204, 2020TRYJ(LB)06, 2020TRYJ(JC)07, TRGG202101, TRYJ2021JC06). They played no role in the study design, data collection or analysis, decision to publish, or manuscript preparation.

Grant number(s)

State the funder, grant or award number and the date of award

13. * Conflicts of interest.

List actual or perceived conflicts of interest (financial or academic).

None

14. Collaborators.

Give the name and affiliation of any individuals or organisations who are working on the review but who are not listed as review team members. **NOTE: email and country must be completed for each person, unless you are amending a published record.**

15. * Review question.

State the review question(s) clearly and precisely. It may be appropriate to break very broad questions down into a series of related more specific questions. Questions may be framed or refined using PI(E)COS or similar where relevant.

The application of radiomics in giant cell tumor of bone (GCTB) for diagnosis, prediction or prognosis.

16. * Searches.

State the sources that will be searched (e.g. Medline). Give the search dates, and any restrictions (e.g. language or publication date). Do NOT enter the full search strategy (it may be provided as a link or attachment below.)

Primary publications concerning radiomics or image texture analysis of CT, MRI, PET/CT or PET/MR in patients with GCTB will be included in this review. Electronic databases including PubMed, Embase, Web of Science, China National Knowledge Infrastructure, and Wanfang Data will be searched. Literature search strategies will be developed using medical subject headings (MeSH) and derived words, including radiomics, textural analysis, CT, MR, PET, GCTB, etc. No restriction will be made regarding publication period. The search strategy will include only terms relating to the review question. Publications must be available in English, Japanese, Chinese, German or French.

17. URL to search strategy.

Upload a file with your search strategy, or an example of a search strategy for a specific database, (including the keywords) in pdf or word format. In doing so you are consenting to the file being made publicly accessible. Or provide a URL or link to the strategy. Do NOT provide links to your search **results**.

https://www.crd.york.ac.uk/PROSPEROFILES/185399_STRATEGY_20220804.pdf

Alternatively, upload your search strategy to CRD in pdf format. Please note that by doing so you are consenting to the file being made publicly accessible.

Do not make this file publicly available until the review is complete

18. * Condition or domain being studied.

Give a short description of the disease, condition or healthcare domain being studied in your systematic review.

Giant cell tumor of bone (GCTB) is one of the most common intermediate bone tumors, which occurs in young adults 20– 40 years old with a high recurrence rate (20%–50%) (1) and a potential for aggressive behavior. Imaging examinations play an important role in diagnosis and differential diagnosis, therapy response evaluation as well as prognosis prediction of GCTB. However, radiological practice relies mainly on the subjective interpretation of imaging data by an expert radiologist and therefore is dependent on reader experience. Quantitative, reader independent analysis, i. e. radiomics model or texture analysis, may supplement expert opinion and improve diagnostic, predictive and prognostic accuracy. This systematic review will study the application of radiomics model or texture analysis in human patients with GCTB.

19. * Participants/population.

Specify the participants or populations being studied in the review. The preferred format includes details of both inclusion and exclusion criteria.

Participants inclusion criteria:

1) patients with histologically confirmed GCTB;

2) patients had undergone at least one pre- treatment pre- or post-treatment CT, MRI, PET/CT or PET/MR;

3) a radiomics model or texture analysis for stratification of tumor, prediction of response to therapy or prognosis of patients was established.

Participants exclusion criteria:

1) not human patients, e. g. cell line, xenotransplant;

2) not GCTB, e. g. osteosarcoma, Ewing sarcoma;

3) no performed imaging procedure;

4) no radiomics model established or texture analysis performed.

20. * Intervention(s), exposure(s).

Give full and clear descriptions or definitions of the interventions or the exposures to be reviewed. The preferred format includes details of both inclusion and exclusion criteria.

Patients with GCTB underwent at least one pre- treatment pre- or post-treatment CT, MRI, PET/CT or PET/MR with a radiomics model or texture analysis performed based on these imaging data.

21. * Comparator(s)/control.

Where relevant, give details of the alternatives against which the intervention/exposure will be compared

(e.g. another intervention or a non-exposed control group). The preferred format includes details of both inclusion and exclusion criteria.

Standard-of-care imaging.

22. * Types of study to be included.

Give details of the study designs (e.g. RCT) that are eligible for inclusion in the review. The preferred format includes both inclusion and exclusion criteria. If there are no restrictions on the types of study, this should be stated.

Studies describing radiomics model or texture analysis of CT, MRI, PET/CT or PET/MR in patients with GCTB for diagnosis purpose, stratification of tumor, prediction of response to therapy or prognosis will be included in this review. Studies must be with full-text available and sufficient information for assessing the methodological quality.

23. Context.

Give summary details of the setting or other relevant characteristics, which help define the inclusion or exclusion criteria.

Study inclusion criteria:

1) studies are reported in English, Japanese, Chinese, German or French with institutional full-text availability;

2) the cohort consists of patients with histologically confirmed GCTB;

3) patients had undergone at least one pre- treatment pre- or post-treatment CT, MRI, PET/CT or PET/MR;

4) a radiomics model or texture analysis for stratification of tumor, prediction of response to therapy or prognosis of patients was established.

Study exclusion criteria:

1) duplicate studies;

2) reviews, technical reports, letters to editors, comments to published studies, conference proceedings, case reports, brief communications and articles with insufficient information for assessing the methodological quality;

3) studies are reported other than English, Japanese, Chinese, German or French;

4) not human, not GCTB, not radiomics or texture analysis studies.

24. * Main outcome(s).

Give the pre-specified main (most important) outcomes of the review, including details of how the outcome is defined and measured and when these measurement are made, if these are part of the review inclusion criteria.

The characteristics of included study will be summarized. The methodological quality, reporting quality, image pre-processing steps, and risk of bias and concern on application, will be assessed.

Measures of effect

Please specify the effect measure(s) for you main outcome(s) e.g. relative risks, odds ratios, risk difference, and/or 'number needed to treat.

The studies will be assessed by Radiomics Quality Score (RQS), Transparent Reporting of a multivariable prediction model for Individual Prognosis Or Diagnosis (TRIPOD) statement, Checklist for Artificial Intelligence in Medical Imaging (CLAIM), and modified Quality Assessment of Diagnostic Accuracy Studies (QUADAS-2) tool.

25. * Additional outcome(s).

List the pre-specified additional outcomes of the review, with a similar level of detail to that required for main outcomes. Where there are no additional outcomes please state 'None' or 'Not applicable' as appropriate to the review

If a sufficient number of studies attempts to answer a similar question, a meta-analysis may be performed to present the performance of those radiomics models.

Measures of effect

Please specify the effect measure(s) for you additional outcome(s) e.g. relative risks, odds ratios, risk difference, and/or 'number needed to treat.

Measures will be made during the data analysis phase.

26. * Data extraction (selection and coding).

Describe how studies will be selected for inclusion. State what data will be extracted or obtained. State how this will be done and recorded.

Study inclusion criteria:

1) studies are reported in English, Japanese, Chinese, German or French with institutional full-text availability;

2) the cohort consists of patients with histologically confirmed GCTB;

3) patients had undergone at least one pre- treatment pre- or post-treatment CT, MRI, PET/CT or PET/MR;

4) a radiomics model or texture analysis for stratification of tumor, prediction of response to therapy or prognosis of patients was established.

Study exclusion criteria:

1) duplicate studies;

2) reviews, technical reports, letters to editors, comments to published studies, conference proceedings, case reports, brief communications and articles with insufficient information for assessing the methodological quality;

3) studies are reported other than English, Japanese, Chinese, German or French;

4) not human, not GCTB, not radiomics or texture analysis studies.

A data collection tool will be established based on similar reviews and then trialed on two randomly chosen studies, which fulfilled all the inclusion criteria. These shall be used to train reviewers to appropriately apply the data extraction tool.

27. * Risk of bias (quality) assessment.

State which characteristics of the studies will be assessed and/or any formal risk of bias/quality assessment tools that will be used.

The risk of bias and quality of studies will be assessed by Radiomics Quality Score (RQS), Transparent Reporting of a multivariable prediction model for Individual Prognosis Or Diagnosis (TRIPOD) statement, Checklist for Artificial Intelligence in Medical Imaging (CLAIM), and modified Quality Assessment of Diagnostic Accuracy Studies (QUADAS-2) tool.

28. * Strategy for data synthesis.

Describe the methods you plan to use to synthesise data. This **must not be generic text** but should be **specific to your review** and describe how the proposed approach will be applied to your data. If meta-analysis is planned, describe the models to be used, methods to explore statistical heterogeneity, and software package to be used.

A narrative synthesis will be provided with information presented in the text and/or tables to summarize and explain the characteristics and findings of the included studies. A quantitative synthesis will be done if the included studies are sufficiently homogenous. All analysis will be based on aggregate data.

29. * Analysis of subgroups or subsets.

State any planned investigation of ‘subgroups’. Be clear and specific about which type of study or participant will be included in each group or covariate investigated. State the planned analytic approach.

If a sufficiently homogeneous subset of studies analyzed a single outcome parameter, e.g. differential diagnosis models based on radiomics or texture analysis, a meta-analysis of this subgroup may be attempted.

30. * Type and method of review.

Select the type of review, review method and health area from the lists below.

Type of review

Cost effectiveness
No

Diagnostic
Yes

Epidemiologic
No

Individual patient data (IPD) meta-analysis
No

Intervention
No

Living systematic review
No

Meta-analysis
No

Methodology

No

Narrative synthesis
No

Network meta-analysis
No

Pre-clinical
No

Prevention
No

Prognostic
Yes

Prospective meta-analysis (PMA)
No

Review of reviews
No

Service delivery
No

Synthesis of qualitative studies
No

Systematic review
Yes

Other
No

Health area of the review

Alcohol/substance misuse/abuse
No

Blood and immune system
No

Cancer
Yes

Cardiovascular
No

Care of the elderly
No

Child health
No

Complementary therapies
No

COVID-19
No

Crime and justice
No

Dental
No

Digestive system
No

Ear, nose and throat
No

Education
No

Endocrine and metabolic disorders
No

Eye disorders
No

General interest
No

Genetics
No

Health inequalities/health equity
No

Infections and infestations
No

International development
No

Mental health and behavioural conditions
No

Musculoskeletal
Yes

Neurological
No

Nursing
No

Obstetrics and gynaecology
No

Oral health
No

Palliative care
No

Perioperative care
No

Physiotherapy
No

Pregnancy and childbirth
No

Public health (including social determinants of health)

No

Rehabilitation
No

Respiratory disorders
No

Service delivery
No

Skin disorders
No

Social care
No

Surgery
No

Tropical Medicine
No

Urological
No

Wounds, injuries and accidents
No

Violence and abuse
No

31. Language.

Select each language individually to add it to the list below, use the bin icon to remove any added in error.
English

There is not an English language summary

32. * Country.

Select the country in which the review is being carried out. For multi-national collaborations select all the countries involved.

China

33. Other registration details.

Name any other organisation where the systematic review title or protocol is registered (e.g. Campbell, or The Joanna Briggs Institute) together with any unique identification number assigned by them. If extracted data will be stored and made available through a repository such as the Systematic Review Data Repository (SRDR), details and a link should be included here. If none, leave blank.

34. Reference and/or URL for published protocol.

If the protocol for this review is published provide details (authors, title and journal details, preferably in Vancouver format)

Add web link to the published protocol.

Or, upload your published protocol here in pdf format. Note that the upload will be publicly accessible.

No I do not make this file publicly available until the review is complete

Please note that the information required in the PROSPERO registration form must be completed in full even if access to a protocol is given.

35. Dissemination plans.

Do you intend to publish the review on completion?

Yes

Give brief details of plans for communicating review findings.?

We planed to publish the systematic review via peer-reviewed journals.

36. Keywords.

Give words or phrases that best describe the review. Separate keywords with a semicolon or new line. Keywords help PROSPERO users find your review (keywords do not appear in the public record but are included in searches). Be as specific and precise as possible. Avoid acronyms and abbreviations unless these are in wide use.

Giant cell tumor of bone, GCTB, radiomics

37. Details of any existing review of the same topic by the same authors.

If you are registering an update of an existing review give details of the earlier versions and include a full bibliographic reference, if available.

38. * Current review status.

Update review status when the review is completed and when it is published.New registrations must be ongoing so this field is not editable for initial submission.
Please provide anticipated publication date

Review_Ongoing

39. Any additional information.

Provide any other information relevant to the registration of this review.

40. Details of final report/publication(s) or preprints if available.

Leave empty until publication details are available OR you have a link to a preprint (NOTE: this field is not editable for initial submission). List authors, title and journal details preferably in Vancouver format.

Give the link to the published review or preprint.
